# Knockdown and inhibition of hippocampal GPR17 attenuates lipopolysaccharide-induced cognitive impairment in mice

**DOI:** 10.1186/s12974-023-02958-9

**Published:** 2023-11-21

**Authors:** Yusheng Liang, Xu kang, Haiwang Zhang, Heng Xu, Xian Wu

**Affiliations:** 1 Anhui Province Key Laboratory of Major Autoimmune Diseases, Anhui Institute of Innovative Drugs, School of Pharmacy, Anhui Medical University, Hefei, 230032 China; 2 Key Laboratory of Anti-Inflammatory and Immune Medicine, Ministry of Education, Hefei, 230032 China

**Keywords:** Alzheimer’s disease, Lipopolysaccharide, Neuroinflammation, GPR17, Cognitive impairment

## Abstract

**Background:**

Previously we reported that inhibition of GPR17 prevents amyloid β 1–42 (Aβ_1-42_)-induced cognitive impairment in mice. However, the role of GPR17 on cognition is still largely unknown.

**Methods:**

Herein, we used a mouse model of cognitive impairment induced by lipopolysaccharide (LPS) to further investigate the role of GPR17 in cognition and its potential mechanism. The mice were pretreated with GPR17 shRNA lentivirus and cangrelor by microinjection into the dentate gyrus (DG) region of the hippocampus. After 21 days, LPS (0.25 mg/kg, i.p.) was administered for 7 days. Animal behavioral tests as well as pathological and biochemical assays were performed to evaluate the cognitive function in mice.

**Results:**

LPS exposure resulted in a significant increase in GPR17 expression at both protein and mRNA levels in the hippocampus. Gene reduction and pharmacological blockade of GPR17 improved cognitive impairment in both the Morris water maze and novel object recognition tests. Knockdown and inhibition of GPR17 inhibited Aβ production, decreased the expression of NF-κB p65, increased CREB phosphorylation and elevated BDNF expression, suppressed the accumulation of pro-inflammatory cytokines, inhibited Glial cells (microglia and astrocytes) activation, and increased Bcl-2, PSD-95, and SYN expression, reduced Bax expression as well as decreased caspase-3 activity and TUNEL-positive cells in the hippocampus of LPS-treated mice. Notably, knockdown and inhibition of GPR17 not only provided protective effects against cholinergic dysfunction but also facilitated the regulation of oxidative stress. In addition, cangrelor pretreatment can effectively inhibit the expression of inflammatory cytokines by suppressing NF-κB/CREB/BDNF signaling in BV-2 cells stimulated by LPS. However, activation of hippocampal GPR17 with MDL-29951 induced cognitive impairment in normal mice.

**Conclusions:**

These observations indicate that GPR17 may possess a neuroprotective effect against LPS-induced cognition deficits, and neuroinflammation by modulation of NF-κB/CREB/BDNF signaling in mice, indicating that GPR17 may be a promising new target for the prevention and treatment of AD.

**Supplementary Information:**

The online version contains supplementary material available at 10.1186/s12974-023-02958-9.

## Highlights

- Exposure to LPS leads to an increase in GPR17 expression in the hippocampus.
- Knockdown and inhibition of GPR17 ameliorates cognitive impairment induced by LPS in mice.
- Knockdown and inhibition of GPR17 protects against neuroinflammation induced by LPS in mice.
- The central GPR17 may be a potential therapeutic target for the prevention and treatment of cognitive dysfunction.

**Supplementary Information:**

The online version contains supplementary material available at 10.1186/s12974-023-02958-9.

## Introduction

Alzheimer’s disease (AD) is a neurodegenerative disease characterized by a decline in cognitive function [1, 2]. Numerous studies have shown that neuroinflammation is well-documented as part of the neuropathogenesis of cognitive deficit [3, 4]. However, the precise pathophysiology of neuroinflammation’s impact on cognition is still not entirely understood.

Lipopolysaccharide (LPS) is an endotoxin derived from bacteria that stimulates the immune system. Several studies have shown that LPS injection increases the production of amyloid β (Aβ), promotes neuroinflammation and oxidative stress, disrupts synaptic function, causes neuronal death, and impairs spatial learning [5–7]. Furthermore, the cognitive impairment induced by LPS may be associated with the dysregulation of the cholinergic system, as seen by a reduction in levels of acetylcholine (Ach), a neurotransmitter involved in the processes of memory and learning [8]. Therefore, LPS injection is an extensively utilized paradigm for exploring the molecular of cognitive deficits. Evidence has also shown that oxidative stress induced by LPS was involved in memory impairment [9]. In addition, LPS induces the activation of nuclear factor kappa B (NF-κB) and the production of pro-inflammatory cytokines [10]. These inflammatory cytokines can cause neuronal damage, leading to the progression of cognitive impairment. Microglia and astrocytes are two important cell types with immune activity in the brain [11]. LPS activates inflammatory cells, such as microglia and astrocytes cells in the brain, which can exacerbate neuroinflammation [12, 13]. Microglia activation causes a malfunction in microglia clearance of Aβ plaques as well as excessive formation [14]. Therefore, inhibition of microglia and astrocytes activation to reduce inflammatory factors is an essential strategy for the treatment of neuroinflammation. In addition, through activating caspase, LPS causes neuroinflammation-mediated neuronal death [15]. Caspase-3 has been identified as an important apoptosis mediator in brain cells [16]. Bax, a pro-apoptotic molecule, causes neuronal cell death, whereas Bcl-2 has the opposite effect [17]. Inhibition of the NF-κB pathway was found to reduce neuroinflammation and apoptosis in response to LPS [18, 19]. The study suggests that NF-κB can directly bind to the promoter region of BACE1 and enhance the transcription of BACE1, which in turn leads to an increase in Aβ production [20]. Neuroinflammation has negative effects on synapses by dissolving or eliminating synapses in hippocampal regions [21]. Numerous studies have shown that LPS impairs memory by modulating NF-κB-mediated brain-derived neurotrophic factor (BDNF) expression and cAMP response element binding protein (CREB) phosphorylation [19, 22]. Therefore, these findings suggest that blocking NF-κB can be beneficial in managing cognitive function by reducing neuroinflammation and apoptosis while improving synaptic function.

G protein-coupled receptor 17 (GPR17) is an orphan receptor that is widely expressed in the central nervous system (CNS) [23] and found in both neurons and microglia cells [24]. Moreover, it has been reported that GPR17 mediates microglia inflammation [25], and its knockout can reduce microglia activation and neuronal damage [26]. According to Zhao et al., an increase in GPR17 expression in neurons has been linked to greater cell damage [27]. However, knockdown of GPR17 can reduce cell injury induced by the agonist leukotriene D4 [26]. In addition, Harrington et al. reported that GPR17 expression is upregulated in several animal models of demyelinating disease [28]. Recent evidence has demonstrated that the blockade of GPR17 would be a promising approach for treating inflammatory-associated diseases, including multiple sclerosis, and brain and spinal cord injury [29]. It has been also reported that GPR17 regulates the NF-κB pathway, and inhibition of this pathway can improve neuroinflammation, oxidative stress, and synaptic dysfunction [30, 31]. Cangrelor, a GPR17 antagonist, has been shown to inhibit GPR17 signaling [25, 32, 33], and reduce pulmonary fibrosis by inhibiting GPR17-mediated inflammation in mice [25]. To date, the effects of GPR17 on LPS-induced cognitive deficits have not been examined. Therefore, in this study, we investigated the role and possible mechanism of GPR17 on cognitive deficits induced by LPS in mice.

## Materials and methods

### Animals

Male Institute of Cancer Research (ICR) mice (male, 6–8 weeks, 22–25 g) were obtained from the Center of Laboratory Animals of Anhui Medical University. Female animals are usually not included, as the release of estrogen may confuse the results. All mice were housed in groups of 3 to 5 per plastic cage and kept under standard conditions with sufficient food and water. The indoor temperature was controlled at 22–25 °C, the humidity was controlled at 40–50%, and the daily lighting time was from 7:00 to 19:00, the light and darkness alternated every 12 h about a week’s adjustment to the environment. All experiments were approved by the experimental animal ethics committee of the Animal Experiment Center, Anhui Medical University.

### Drugs and reagents

Cangrelor was obtained from Cayman Chemical (Ann Arbor, USA). MDL-29951 was from Med Chem Express. LPS (Escherichia coli serotype 0111:B4) was from Sigma-Aldrich (St. Louis, USA). Antibodies were from several companies: anti-GPR17 was from Servicebio (Wuhan, China); anti-APP, anti-BACE1, anti-BDNF, and anti-CREB were from Abcam (Cambridge, USA); anti-NeuN, anti-Iba1, anti-GFAP, anti-postsynaptic density protein 95 (PSD-95), anti-synaptophysin (SYN), and anti-β-actin were from Cell Signaling Technology, Inc (Massachusetts, USA). CD68 was from Proteintech (Wuhan, China). Anti-NF-κB p65, anti-caspase-3, anti-Bcl-2, anti-Bax were from Affinity Biosciences Co., Ltd (Jiangsu, China). Anti-Histone H3 was from Bioworld Technology Co., Ltd (Minneapolis, USA). Goat anti-rabbit or anti-mouse IgG-HRP (Proteintech, China) was used as the secondary antibody. The nucleoprotein extraction kit was from Sangon Biotech Co., Ltd (Shanghai, China). Other general agents are purchased from commercial suppliers. The ChAT and Ach assay kits were purchased from Nanjing Jiancheng Bioengineering Institute (Nanjing, China). The AchE assay kit was purchased from Solarbio (Beijing, China).

### LPS-induced cognitive impairment mouse model

We mainly referred to previous literature to induce animal models of cognitive impairment by LPS. All groups except the control were intraperitoneal (i.p.) injection of LPS (0.25 mg/kg) for 7 days with the control group receiving i.p. injection of the same volume of physiological saline [34]. After LPS administration, some animals were subjected to behavioral testing, while others were subjected to molecular biological testing.

#### Lentivirus-mediated knockdown experiment

Mice were randomly divided into three groups (*n* = 12 mice/group): Vehicle + Vehicle (Veh + Veh or control), LV-EGFP + Lipopolysaccharide (LV-EGFP + LPS), LV-GPR17–shRNA–EGFP + Lipopolysaccharide (LV-GPR17–shRNA–EGFP + LPS). The dura was exposed and a microinjection pump was used to bilaterally infuse the LV-EGFP or LV-GPR17–shRNA–EGFP (1.0 μl/side at an injecting rate of 0.20 µl/min) using the following coordinates: 2.0 mm caudal to bregma, 1.5 mm from the midline, and 2.0 mm below the dural surface [34]. After 21 days, the mice were given a systemic injection of LPS (0.25 mg/kg, 0.1 ml/10 g of body weight) for 7 days, and then subjected to behavioral tests followed by a series of histopathological/biochemical tests (Fig. 2A).

#### The drug treatment experiment

The first part is a pharmacological antagonism experiment. Mice were randomized into four groups (*n* = 12 mice/group): Group I: Vehicle + Vehicle (Veh + Veh or control); Group II: vehicle plus LPS (Veh + LPS); Group III: cangrelor 2.0 μg plus LPS (cangrelor 2.0 μg + LPS); Group IV: cangrelor plus LPS (cangrelor 4.0 μg + LPS). The second part is the pharmacological activation experiment. Three groups were used (*n* = 12 mice/group): Group I: Vehicle + Vehicle (Veh + Veh or control); Group II: MDL-29951 plus vehicle (MDL-29951 2.0 μg + Veh); Group III: MDL-29951 2.0 μg plus LPS (MDL-29951 2.0 μg + LPS). LPS solution was prepared with 0.9% NaCl saline, and the control group was intraperitoneally injected with 10 ml/kg saline. A detailed schedule of treatment and behavioral tests is shown in Figs. 3A and 8A. A cannula was surgically implanted into the hippocampal DG region using the following coordinates: 2.0 mm caudal to bregma, 1.5 mm from the midline, and 2.0 mm below the dural surface (RWD Life Science, Shenzhen, China). We removed the dummy cannula and inserted the internal infusion needles (O.D.: 0.21 mm; RWD, Shenzhen, China) into the cannula guides. The head of the cannula was connected to the PE tubes (O.D.: 0.50 mm). After 1 week of recovery, cangrelor or MDL-29951 was infused into the hippocampal DG. The mice were administered with cangrelor or MDL-29951 every day for 3 weeks [34].

### Lentivirus generation and microinjection

GPR17 was silenced by lentiviral shRNA-mediated knockdown. We generated a lentiviral vector construct expressing short hairpin RNA (shRNA) complementary to the coding exon of the mice GPR17 gene and tagged it with a fused enhanced green fluorescent protein (EGFP) and named it LV-GPR17–shRNA–EGFP (viral titer, 3 × 10^8^ TU/ml). There was also a non-silencing shRNA control (LV-shRNA–EGFP) created. The sequences for scrambled control-shRNA and GPR17–shRNA were 5′-TTCTCCGAACGTGTCACGT-3′ and 5′-CCGGATAGAGAAGCACCTCAA-3′, respectively (Hanbio, Shanghai, China). For the virus infusion, the animals were anesthetized with isoflurane, and 2.0 μl control-shRNA (1.0 μl/side) or GPR17–shRNA–EGFP (1.0 μl/side) were injected into the hippocampus. Then, the animals were returned to their home cages while being kept warm. After 3 weeks of recovery, the mice were intraperitoneally injected with LPS (0.25 mg/kg) or its vehicle for a week, and 24 h after the last LPS administration, immunofluorescence, Western blotting, and RT-PCR were used to observe the transfection effect in some mice (*n* = 4 mice/group), and behavioral experiments were performed in other animals (*n* = 12 mice/group).

### Cell culture and drug treatment

BV-2 cells, a model of microglia, were maintained in complete DMEM (containing 1% antibiotics and 10% FBS). The cells were incubated in a humidified environment of 5% CO_2_ at 37 °C. All experiments were carried out the day after the cells were seeded in 96-well culture plates. To stimulate an inflammatory response, the culture medium was replaced with fresh DMEM (including 1% antibiotics), and 1 μg/ml of LPS was added in the presence or absence of cangrelor (20, 40, and 80 μM). After drug treatment, the protein is extracted for analysis.

BV-2 cells were transfected with GPR17 siRNA (Hanbio, Shanghai, China) or control siRNA for the GPR17 knockdown experiment. In brief, 6 µl siRNA was combined with 12 µl transfection reagents in 500 µl serum-free medium for 30 min. When the transfection reagent was added to a 60 mm petri dish, the cell fusion rate was about 50%. After transfection, the cells were allowed to grow for another 48 h. GPR17 knockdown efficiency was determined by Western blot analysis.

### CCK‐8 cell viability assay and ELISA for inflammatory cytokine secretion

To determine cell viability and cytokine secretion, cells were plated in 96-well culture plates (5 × 10^5^ cells/ml) and were incubated with different concentrations of cangrelor (20, 40, and 80 μM) and 1 μg/ml LPS for 24 h (for ELISA) or 48 h (for viability assay). To assess cell viability, 10 μl CCK-8 solutions were added to each well and then incubated at 37 °C for an additional 1 h. The absorbance at 450 nm was determined using a microplate reader. For the ELISA, the supernatant of BV-2 cells was collected through centrifuging at 300 × g for 10 min. The levels of TNF-α, IL-1β, and IL-6 were measured via ELISA kits (ABclonal) according to the manufacturer’s protocol.

### Behavioral testing

#### Open field test (OFT)

Locomotor activity was investigated using published approaches [35]. The experiment was conducted for 2 days. The first day of the adaptation phase and the second day of the testing phase. The mice’s locomotor activity was recorded for 5 min in a locomotor monitoring device (50 cm × 50 cm × 35 cm). The open field was cleaned with 5% ethyl alcohol and allowed to dry between tests.

#### Morris water maze (MWM) test

The MWM test is one of the most widely used experiments to study the behavioral neuroscience of spatial learning and memory [36]. MWM test consisted of three successive trials (visible-platform, hidden-platform, and spatial probe trial). The MWM consisted of a large circular black pool (120-cm diameter, 60-cm height) filled with water at 24 ± 2 °C. The maze was divided into four quadrants, and a clear platform was placed inside the water (hidden 1 cm). A mounted flag (height, 5 cm) was fixed to the platform throughout the visible platform trial (days 1–2). Each mouse was subjected to four training sessions every day in both the visible platform and the hidden platform (days 3–5). They had 90 s to seek the platform, and if they did not find the platform within 90 s, they would be directed to it by the experimenter and stay there for 10 s. In the probe test (day 6, without the flag attached), the mice were allowed to swim in the water tank for 90 s without the hidden-platform. The trend of the mouse searching for the platform was measured by the time spent in the target quadrant, where the platform was previously located. The number of crossings on the target platform was recorded by video tracking equipment and processed by a computer equipped with an analysis-management system.

#### Novel object recognition (NOR) test

NOR test is widely used to test memory, as described previously [36]. Mice were placed in the center of the open field chamber (50 cm × 50 cm × 35 cm). Mice completed an acquisition session in which they were left in an apparatus containing two similar objects. 24 h later, recognition memory was assessed and a different pair of dissimilar objects (a familiar and a novel one, respectively) were presented. Time spent exploring both objects was recorded for 5 min.

### Western blot analyses

Hippocampal tissue or BV-2 cells were homogenized in a mixture of PMSF and RIPA lysis buffer (Beyotime Institute of Biotechnology, Shanghai, China) on ice. Supernatant protein concentrations were determined after centrifugation at 12,000 rpm for 5 min at 4 °C with a BCA protein assay kit (Beyotime Institute of Biotechnology, Shanghai, China). Protein samples (30–40 μg) were added to the band, fractionated by 6–12% sodium dodecyl sulfate–polyacrylamide gels (SDS–PAGE), and transferred to polyvinylidene difluoride (PVDF) membrane (0.22 μm; Millipore, Temecula, CA, USA). Subsequently, membranes were blocked in 5% non-fat milk for 2 h and probed with primary antibodies overnight at 4 °C. The primary antibodies used in this study were anti-GPR17 (1:300), anti-APP (1:500), anti-BACE1 (1:500), anti-Iba1 (1:1000), anti-GFAP (1:1000), anti-CD68 (1:1000), anti-caspase-3 (1:1000), Bcl-2 (1:1000), Bax (1:500), anti-BDNF (1:1000), and anti-β-actin (1:2000), respectively, followed by incubation with horseradish peroxidase (HRP)-conjugated goat anti-rabbit or goat anti-mouse secondary antibody (1:8000, Proteintech, China) at room temperature for 2 h. Nuclear proteins were extracted using nucleoprotein extraction (Sangon Biotech, China). The supernatant nuclear protein extract was submitted to Western blot for assays of anti-NF-κB p65 (1:1000), anti-CREB (1:1000), and anti-Histone H3 (1:500). Finally, the protein bands were visualized using an enhanced chemiluminescence kit (Millipore, Billerica, MA, USA). The images were digitized from the membrane and the band intensity was quantified using Image J software, version 2.0 (*n* = 4 mice/group).

### Extraction of nuclear protein

Nuclear extract was prepared using a nucleoprotein extraction kit. Briefly, the mouse hippocampus was chopped into small pieces, homogenized in ice-cold hypotonic buffer containing 0.5% phosphatase inhibitor, 1% PMSF, and 0.1% DTT, and then centrifuged at 4 °C, 3000 g for 5 min. The precipitate was washed with hypotonic buffer and centrifuged at 4 °C, 5000 g for 5 min. Finally, 0.2 ml lysis buffer containing 0.5% phosphatase inhibitor, 1% PMSF, and 0.1% DTT were added into the precipitate, chilled for 20 min, and centrifuged at 4 °C, 15,000 g for 10 min. The supernatant nuclear protein extract was subjected to WB for assay of NF-κB p65, p-CREB, and histone H3 was used as a loading control (*n* = 4 mice/group).

### Real-time quantitative PCR (RT-PCR) analysis

GPR17 mRNA level in the mouse hippocampus was assessed by RT-PCR. Mice were sacrificed after the behavioral analysis, and the brains were rapidly removed. The mRNA from the mouse hippocampus was isolated and purified by standard techniques using Trizol as per the manufacturer’s protocol (Invitrogen, USA). The mRNA was reverse transcribed using Superscript II reverse transcriptase (Invitrogen, USA), and an aliquot of the reaction was used in simultaneous PCR reactions. A BioRad MyIQ real-time thermocycler was used to collect the data. For PCR, SYBR green detection was used according to the manufacturer’s protocol (Bio-Rad). Amplicon identity was checked by restriction analysis. The primer efficiency was determined from the slope relation between the absolute copy number or RNA quantity and the cycle threshold using the BioRad software. All primer pairs had a minimum of 90% efficiency. Primers that amplify β-actin mRNA were used as a control to normalize the data. Primer sequences comprised: β-actin Forward: GGCTGTATTCCCCTCCATCG Reverse: CCAGTTGGTAACAATGCCATGT; GPR17 Forward: CACCCTGTCAAGTCCCTCAAG Reverse: GTGGGCTGACTAGCAGTGG. The data of real-time PCR were analyzed using the value 2−^△△Ct^. β-Actin was used as an internal control (*n* = 4 mice/group).

### Determination of the cholinergic system, cytokines release, and Aβ

Acetylcholine esterase (AChE) is a marker for the imbalance in the cholinergic system and the loss of cholinergic neurons in the brain. The AChE activity was assessed using an AChE ELISA kit. Briefly, take 50 μl of the supernatant from the hippocampal tissue homogenate and mix it with 3 ml of sodium phosphate buffer, 100 μl of the acetylthiocholine iodide, and 100 μl of Ellman’s reagent. Measure the change in absorbance by spectrophotometry at 412 nm (Solarbio, China). Furthermore, acetylcholine (ACh) and choline acetyltransferase (ChAT) levels in the hippocampus were evaluated by corresponding kits according to the manufacturer’s protocol (Nanjing Jiancheng Biological Engineering Institute, Nanjing, China). The homogenate was centrifuged and the supernatant was collected carefully and assayed by TNF-α, IL-1β, and IL-6 ELISA kit (ABclonal) according to the procedures supplied by the manufacturer. The level of Aβ in the hippocampus was also determined using ELISA kits (*n* = 4 mice/group).

### Immunofluorescence staining

The mouse brain tissue was post-fixed in the perfusion solution for 4 h at 4 °C before being incubated in a 30% sucrose solution for 24 h. The Tissue-Tek O.C.T. compound (Sakura, Japan) was used to embed brain tissue, which was consequently cut into 20-μm slices (Leica, Germany). The brain slices were soaked in 3% H_2_O_2_ for 30 min, and permeabilized with 0.5% Triton X-100 for 10 min before being blocked with 5% bovine serum albumin (BSA) for 1 h and incubated at 4 °C overnight with anti-Iba1 (1:200), and anti-GFAP (1:200) primary antibody. Following washing, the sections were incubated for 1 h at room temperature in the dark with Alexa Fluor 488 donkey anti-rabbit IgG (1:200; Yeasen Biotech, Shanghai, China) or Alexa Fluor 594 goat anti-mouse IgG (1:200; Yeasen Biotech, Shanghai, China) secondary antibodies. The nuclei were shown by DAPI staining. Photomicrographs were obtained by Leica microsystems (Wetzlar, Germany) and quantified with the Image-Pro Plus software. The number of microglia and astrocytes in the hippocampus was measured, followed by the microglia and astrocytes-positive area to generate the ratio of microglia and astrocytes staining to hippocampal area (% area occupied). The average values of the four slices from each animal were used for statistical analysis (*n* = 4 mice/group).

### The terminal transferase biotinylated-dUTP nick end labeling (TUNEL)-staining

TUNEL-staining was performed to label cells undergoing apoptosis following the manufacturer’s instructions (Solarbio, China). Briefly, the brain sections were incubated in a permeabilization solution and then incubated with a TUNEL reaction mixture. Finally, the sections were incubated with TdT enzyme containing FITC-labeled dUTP at 37 °C for 60 min in a humidified dark chamber. After washing with PBS, the sections were stained with DAPI. TUNEL^+^ cells were identified by the co-localization of both the TUNEL signal and DAPI. The apoptotic bodies were expressed as a percentage of the total number of cells examined the percentage of apoptotic cells in the brain sections. Nerve cell-specific immunofluorescence staining marker NeuN was co-located with the TUNEL-positive nucleus. The number of apoptotic neural cells per view was counted using microscopy at × 200 magnification (*n* = 4 mice/group).

### Statistical analysis

Statistical analyses were performed using SPSS v20.0 software. Data were assessed for normality and homogeneity of variance using Kolmogorov–Smirnov and Bartlett’s tests, respectively. Results are expressed as mean ± standard error of the mean (SEM). Comparisons between the two groups were made using Student’s *t* test. The behavioral data of escape latency from MWM were analyzed using two-way analysis of variance (ANOVA) and individual means were compared using post-hoc Bonferroni’s multiple comparison test. All other data were analyzed by a one-way ANOVA followed by Dunnett’s post-hoc analysis for multiple comparisons. All graphs were made using Graph Pad Prism software (version 7.0, Prism Software for PC, GraphPad). Values of *P* < 0.05 were considered statistically significant.

## Results

### GPR17 is upregulated in the mouse brain after LPS exposure

**Fig. 1:**
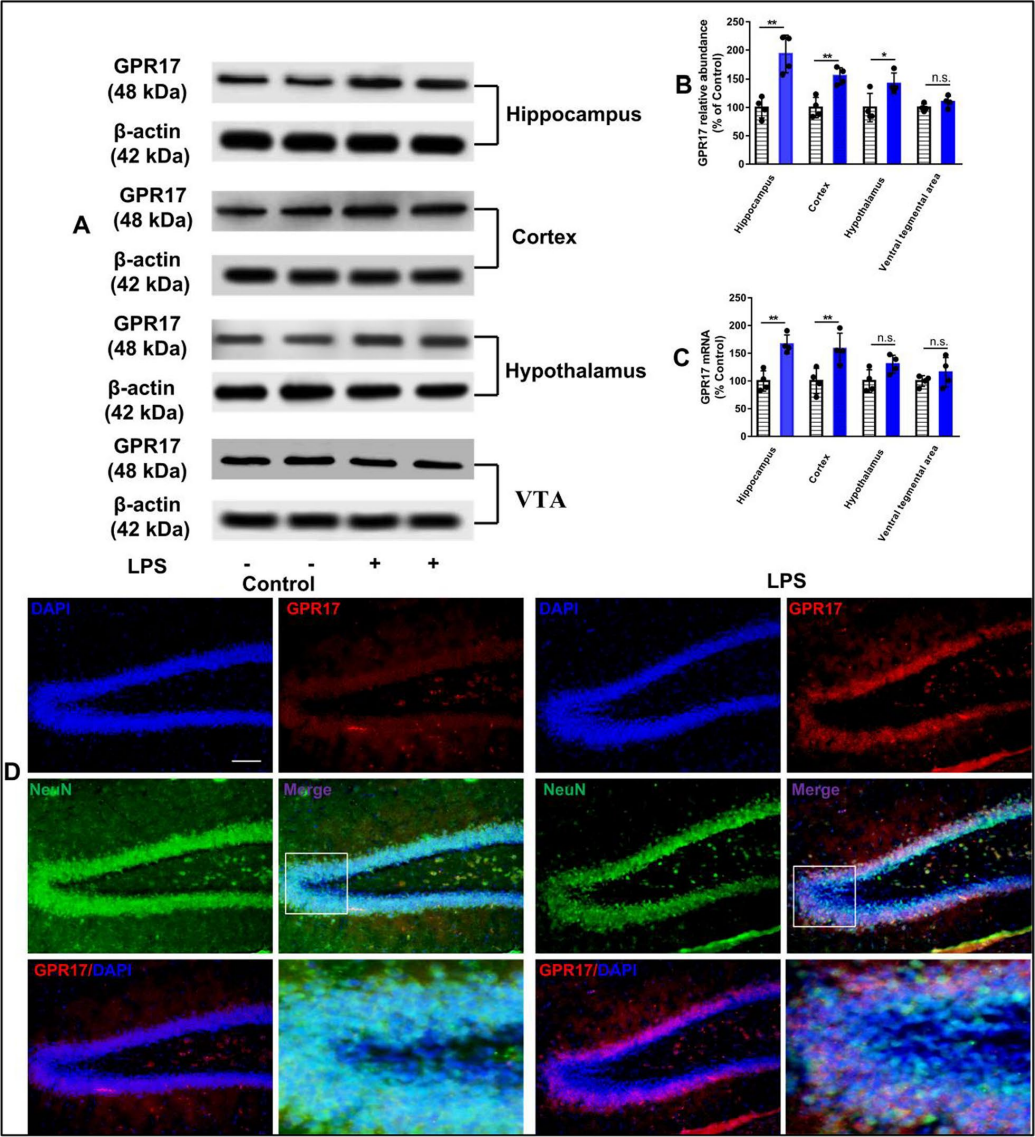
LPS exposure increases GPR17 expression in the mouse brain. The mice were injected with LPS (0.25 mg/kg, i.p.) for 7 days, and the expression of GPR17 in the brain was detected by Western blot, RT-PCR, and immunofluorescent staining after injection. **A** Representative GPR17 protein bands in the hippocampus, cortex, hypothalamus, and VTA were detected after LPS exposure. Quantification of GPR17 protein (**B**) and mRNA (**C**) levels in the brain. **D** Representative images of immunofluorescent staining of GPR17 (red), NeuN (green), and DAPI (blue) in the hippocampal DG from saline mice and LPS mice. Scale bar, 100 μm. All statistics were conducted using a paired *t* test to account for matched control and LPS-treated mice. Values shown are expressed as mean ± SEM; *n* = 4 mice/group. *P < 0.05, **P < 0.01 versus LPS

Since GPR17 is associated with inflammation, we first investigated whether exposure to LPS, an inflammation inducer, can change the expression of GPR17 in the mouse brain. We detected the protein and mRNA levels of GPR17 in the hippocampus, cortex, hypothalamus, and ventral tegmental area (VTA) of the LPS-treated mice. Our results showed that LPS treatment induced a significant upregulation of GPR17 protein in the hippocampus (193.21% ± 16.67%), cortex (154.65% ± 7.14%), and hypothalamus (142.07% ± 8.80%) compared with the control group. However, there was no significant change of GPR17 protein in the VTA (hippocampus: t(6) = 4.90, *P* < 0.01; cortex: t(6) = 4.75, *P* < 0.01; hypothalamus: t(6) = 2.74, *P* < 0.05; VTA: t(6) = 1.60, *P* > 0.05; Fig. 1B). The RT-PCR results further indicated that the mRNA levels of GPR17 was significantly increased in the hippocampus and cortex of the LPS-treated mice compared with the control group, but not in the hypothalamus and VTA (hippocampus: t(6) = 5.31, *P* < 0.01; cortex: t(6) = 3.22, *P* < 0.05; hypothalamus: t(6) = 2.32, *P* > 0.05; VTA: t(6) = 1.09, *P* > 0.05; Fig. 1C). The DG, a subregion of the hippocampus, is thought to be an important brain region for learning and memory. Immunofluorescence results showed that LPS treatment also induced obvious upregulation of GPR17 in the hippocampal DG region (Fig. 1D). Therefore, we chose the hippocampal DG as the target region to further investigate the role of GPR17 in cognitive impairment induced by LPS in mice.

### Knockdown of hippocampal GPR17 prevents LPS-induced cognitive impairment in mice

**Fig. 2:**
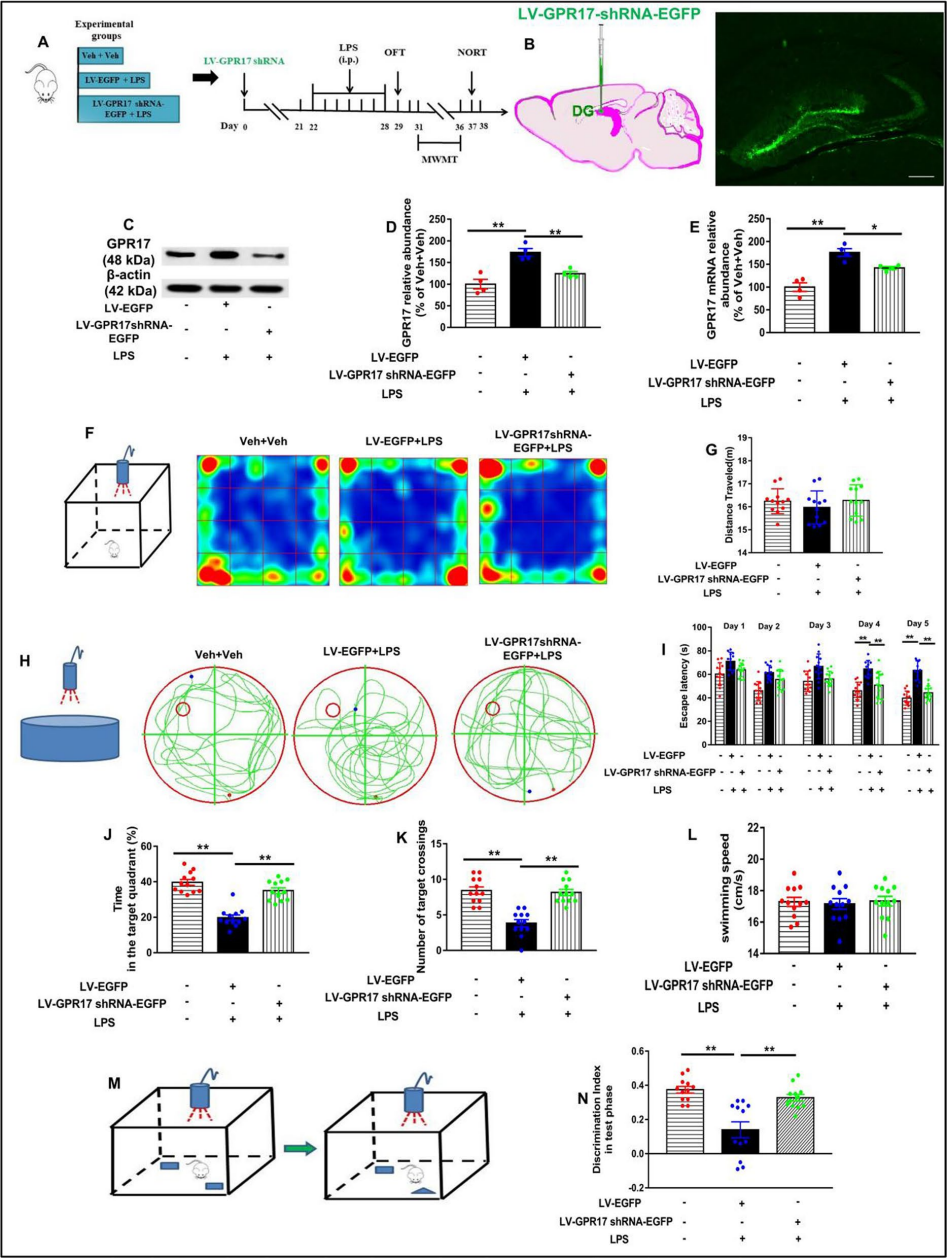
Hippocampal GPR17 knockdown prevents LPS-induced cognitive impairment in mice. **A** Experimental procedure for the test schedule. LV-EGFP or LV-GPR17–shRNA–EGFP were microinfused into bilateral DG regions of the hippocampus of mice, followed by LPS (0.25 mg/kg, i.p.) or its vehicle was administered 7 days after the viral infusions 3 weeks, and then, the OFT, MWM, and NORT tests were conducted in mice. **B** Imaging of green fluorescent protein expression in the hippocampus after 4 weeks of viral vector injection. Scale bar = 100 μm. **C** Representative immunoreactive bands of GPR17 protein in the hippocampus. β-Actin was used as an internal control, and relative protein levels were quantified by densitometry analysis using Image J software. Quantification of GPR17 protein (**D**) and mRNA (**E**) levels in the hippocampus was shown, *n* = 4 mice/group. Scale bar, 100 μm. **F** Schematic diagram of experimental apparatus and the heat map of mouse movement in the OFT. **G** Total distance in the OFT. **H** Representative trajectories of mice from each experimental group in the probe trial. **I** During the 2-day visible platform test, there were no differences in the escape latency among all groups in the MWM test. Escape latency was changed on the hidden platform during the 3-day acquisition trials. **J** Time spent in the target quadrant during the probe trial test. **K** Number of platform crossings during the probe trial test. **L** Swimming speed among all groups during probe testing on day 6. **M** Schematic diagram of experimental apparatus of the NORT. **N** Discrimination index of the NORT. Values shown are expressed as mean ± SEM; *n* = 12 mice/group. *P < 0.05, **P < 0.01 versus Veh + LPS

To verify the role of GPR17 in LPS-induced cognitive dysfunction, we constructed a lentivirus vector carrying GPR17 with EGFP, named LV-GPR17–shRNA–EGFP. To produce a stable knockdown of the hippocampal GPR17, the mice were intrahippocampally injected with LV-GPR17–shRNA–EGFP. On week 4, the EGFP^+^ cells were observed under a fluorescence microscope. Numerous of EGFP^+^ cells were found expressed throughout the hippocampal DG (Fig. 2B). In addition, we also found that EGFP^+^ cells were expressed on microglia (Additional file 1: Fig. S1). Next, we investigated whether the protein and mRNA levels of GPR17 were altered in the hippocampus of LPS-treated mice. Western blotting analysis revealed a significant increase of GPR17 protein in the hippocampus of LPS-treated mice (*F *[2, 9] = 17.96*, P* < *0.01*; Fig. 2C, and D). The RT-PCR results also showed that the expression of the GPR17 gene was upregulated after LPS exposure (*F *[2, 9] = 24.65*, P* < *0.01*; Fig. 2E). These results indicate that LPS treatment induces a uniform increase in hippocampal GPR17 expression. However, LV-GPR17–shRNA–EGFP microinjection into the DG region of the hippocampus significantly decreased the mRNA and protein levels of GPR17 in the DG region of the hippocampus compared with the LV-EGFP lentivirus pretreatment group, indicating the effectiveness of this lentivirus (Fig. 2D, and E).

To investigate the role of GPR17 in cognitive dysfunction induced by LPS, we performed several behavioral tests in mice. Before the MWM and NOR tests, general behavioral performance was assessed by the open field test. As shown in Fig. 2F, G, one-way ANOVA showed no significant difference in the distance of movement among different groups (*F *[2, 33] = 0.80, *P* > 0.05, Fig. 2G), suggesting that the impaired performance in behavioral tests among the groups were not due to the differences in spontaneous locomotor activity. Repeated administration of LPS in mice for 7 days causes memory decline as observed by different behavioral studies. Herein, we employed to test the memory retention ability in the mice under experiment through MWM and NOR tests. During the visible platform training of the MWM test, no significant difference in escape latency was observed among all the groups, suggesting no visual or physical disability in the animals (Fig. 2I). During the non-visible platform training (days 3–5), the escape latency was significantly increased in the LPS (only)-treated group compared to the control group, while the escape latency was significantly reduced in the LV-GPR17–shRNA–EGFP-treated group (Fig. 2I). On day 6 of the MWM test, during the probe trial, the percentage of the total time spent in the target quadrant and the number of platform crossings were considered to evaluate memory retention in the animals. Time spent in the target quadrant (1 trial/mouse, *F *[2, 33] = *20.56, P* < *0.01*, Fig. 2J) and the number of platform crossings (1 trial/mouse, *F *[2, 33] = *22.56, P* < *0.01*, Fig. 2K) were significantly less in the LV-EGFP + LPS group compared with the Veh + Veh group, suggesting memory impairment in the LPS-treated mice. However, LV-GPR17–shRNA–EGFP-treated mice spent more time in the target quadrant (*P* < *0.01*; Fig. 2J), and crossed the platform position more frequently (*P* < *0.01*, Fig. 2K) than the LPS-treated mice. No significant difference was noted in the swimming speed between the three groups (*F *[2, 33] = *0.09, P* > *0.05*, Fig. 2L).

To supplement the results observed in the MWM task, we also carried out the NOR test. The NOR test was used to assess the recognition memory in our study. Due to the innate preference for novelty, if the mice recognized the familiar objects they had seen in the environment, they would spend more time exploring the new object. A significantly decreased recognition index was observed in mice in the LPS group compared with the control group (*F *[2, 33] = *5.29, P* < *0.01*, Fig. 2N), while it was increased in the GPR17–shRNA + LPS group (*P* < *0.01*, Fig. 2N). In addition, GPR17 knockdown in normal mice had no significant effect on their learning and memory (Additional file 1: Fig. S2). Taken together, these results suggest that the knockdown of GPR17 ameliorates LPS-induced spatial and learning impairment in mice.

### Inhibition of GPR17 with cangrelor attenuates cognitive impairment induced by LPS in mice

**Fig. 3:**
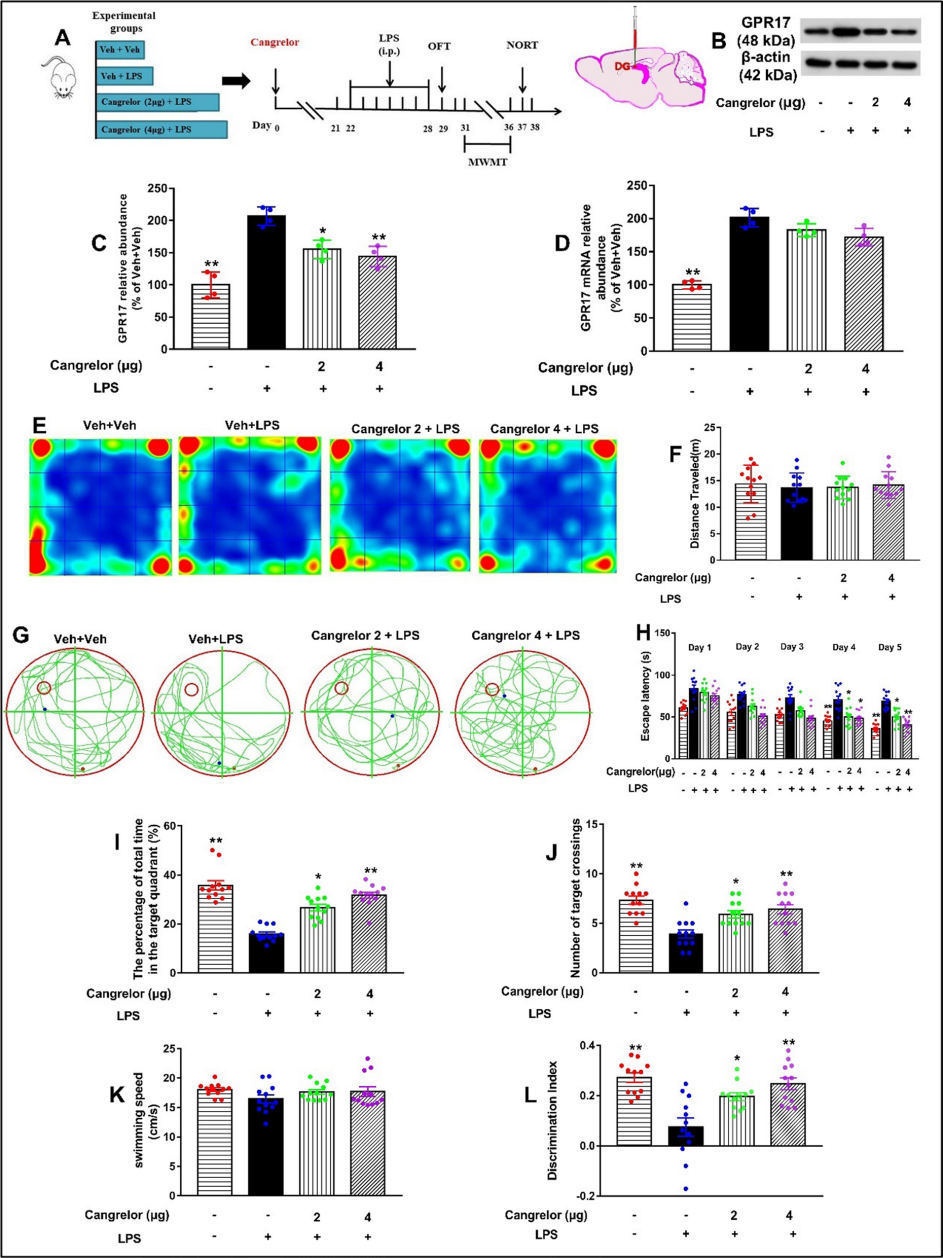
Inhibition of GPR17 with cangrelor attenuates cognitive impairment induced by LPS in mice. Cangrelor was microinfused into bilateral DG regions of the hippocampus of mice for 21 days, followed by LPS (0.25 mg/kg, i.p.) or its vehicle was administered for 7 days, and then, the OFT, MWM, and NORT tests were conducted in mice. **A** Schematic representation of the drug treatment experiment design. **B** Representative immunoreactive bands of GPR17 protein in the hippocampus. GPR17 protein (**C**) and mRNA levels (**D**) in the hippocampus. β-Actin was used as an internal control, *n* = 4 mice/group. **E** Representative heat map of mouse movement in the OFT. **F** Total distance in the OFT was shown. **G** Representative trajectories of mice from each experimental group in the probe trial. **H** During the 2-day visible platform test, there were no differences in the escape latency among all groups. Escape latency was changed on the hidden platform during the 3-day acquisition trials. **I** The percentage of total time spent in the target quadrant during the probe trial. **J** Number of platform crossings during the probe trial test on day 6. **K** Average swimming speed among all groups in the 6 day tests. **L** Discrimination index was determined by performing the following calculation: (EB − EA)/(EB + EA). Values shown are expressed as mean ± SEM; *n* = 12 mice/group. *P < 0.05, ** P < 0.01 versus Veh + LPS

To further verify the role of GPR17 in the regulation of cognitive impairment induced by LPS, we subsequently explored the effects of cangrelor, a GPR17 antagonist, in mice exposed to LPS. Consistent with previous findings, our results showed that hippocampal GPR17 expression was also significantly increased after LPS exposure. Interestingly, cangrelor pretreatment reduced the expression of GPR17 protein (*F *[3, 12] = 29.33, *P* < 0.01, Fig. 3B, and C), but did not affect mRNA levels in the hippocampus (*F *[3, 12] = 33.43, *P* < 0.01, Fig. 3D).

The effects of cangrelor on LPS-induced cognitive deficits in mice were evaluated by behavioral tests. Similar to the virus administration experiment, there were no significant differences in the total distance of the four groups in the OFT (*F *[3, 44] = 0.18 *P* > 0.05, Fig. 3F). This result indicated that cangrelor did not affect the spontaneous locomotor activity in mice. As shown in Fig. 3H, mice in each group exhibited similar escape latency in the visible-platform variant (days 1–2) of the MWM test (Fig. 3H). Then, we tested the mice in the spatial hidden-platform variant (days 3–5). The results showed that the escape latency in the LPS group was significantly longer than that in the control group on days 4 and 5 after LPS injection, which was reversed by cangrelor pretreatment, suggesting that cangrelor ameliorated the spatial learning deficits induced by LPS in mice (Fig. 3H). During the spatial probe test phase (on day 6), mice in the LPS group showed a shorter time spent in the target quadrant (*F *[3, 44] = 27.55, *P* < 0.01, Fig. 3I), and fewer platform crossings (*F *[3, 44] = 11.79, *P* < 0.01, Fig. 3J) compared to the control group, indicating poorer memory for the former platform location. However, pretreatment with cangrelor significantly increased both the percentage of time spent in the target quadrant and the number of platform location crossings compared to the Veh + LPS group (Fig. 3I, J). In addition, no significant difference was noted in the swimming speed between the four groups (*F *[3, 44] = 1.36, *P* > *0.05*, Fig. 3K).

The NOR test was used to determine the time spent on the novel object compared to the familiar object. The DI was calculated as mentioned earlier and shown in Fig. 3L reveals that the group that received LPS alone showed negative DI values, indicating non-spatial memory impairment, where the mice spent more time exploring the familiar object than the novel object (*F *[3, 44] = 12.71, *P* < 0.01, Fig. 3L). Notably, pretreatment with cangrelor showed a significant increase in DI compared with the Veh + LPS group (Fig. 3L). In addition, pharmacological inhibition of GPR17 in normal mice had no significant effect on their learning and memory (Additional file 1: Fig. S3). These data suggest that inhibition of GPR17 with cangrelor improves LPS-induced memory impairment in mice.

### Knockdown and inhibition of hippocampal GPR17 inhibit the production of amyloidogenesis and oxidative response in LPS-treated mice

**Fig. 4:**
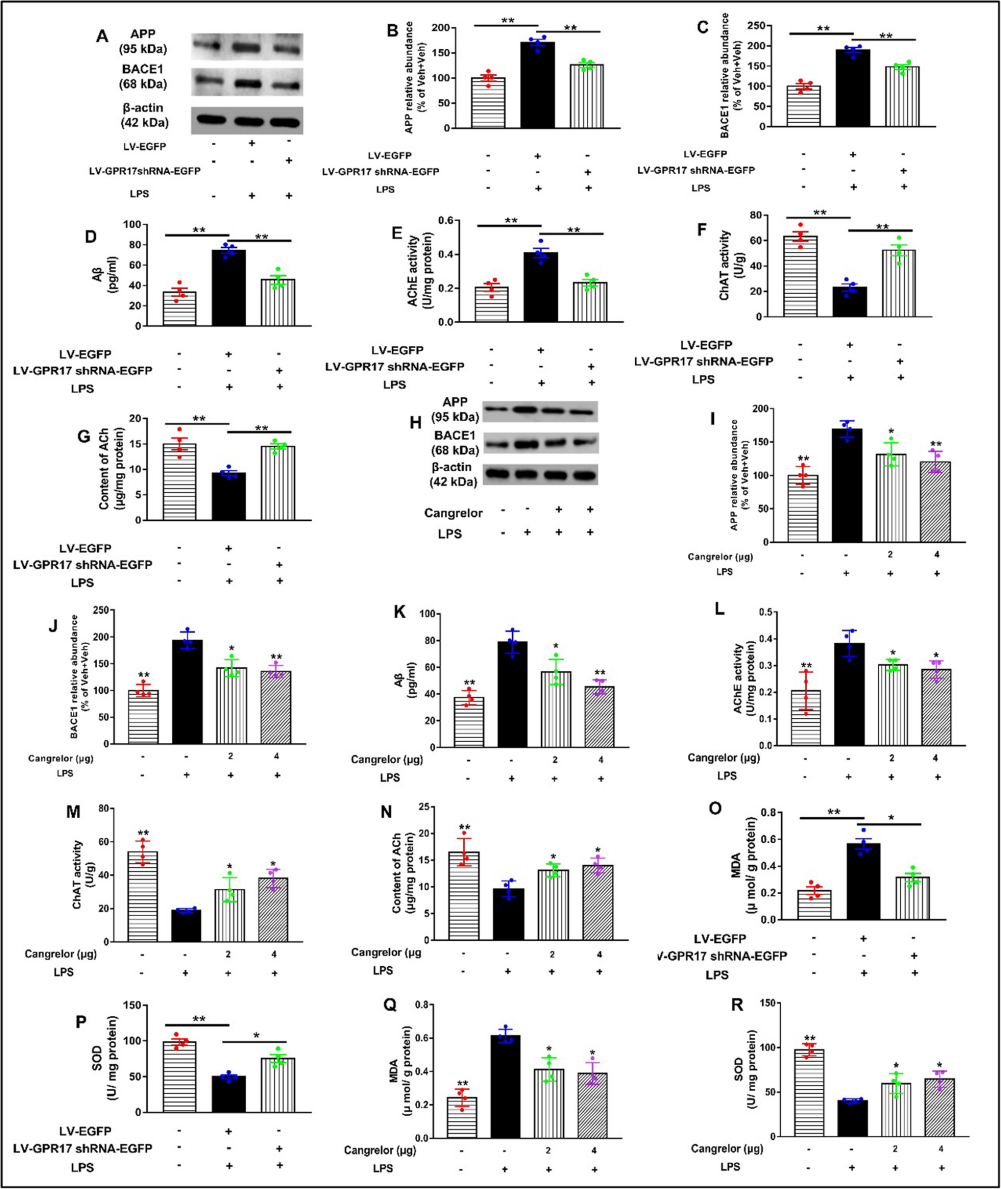
Knockdown and inhibition of hippocampal GPR17 inhibits the production of amyloidogenesis and oxidative response in LPS-treated mice. **A** Representative protein bands of APP and BACE1 in the hippocampus of lentivirus groups were shown. β-Actin was used as an internal control, and relative protein levels were quantified by densitometry analysis using Image J software. Quantification of the changes of APP (**B**) and BACE1 (**C**) protein in the hippocampus were presented as the ratio (in percentage) of the Veh + Veh group. **D** Levels of Aβ in the brain. **E** AchE activity, **F** ChAT activity, and levels of Ach **G** in the mouse brain. **H** Representative images of APP and BACE1 in the hippocampus of cangrelor groups were shown. Quantification of APP (**I**) and BACE1 (**J**) were presented as the ratio (in percentage) of the Veh + Veh group. **K** Levels of Aβ in the brain. **L** AchE activity, **M** ChAT activity, and levels of Ach **N** in the mouse brain. The levels of MDA (**O**) and SOD (**P**) in the hippocampus of lentivirus groups were shown. The levels of MDA (**Q**) and SOD (**R**) in the hippocampus of cangrelor groups were shown. Data shown are expressed as mean ± SEM; *n* = 4 mice/group. *P < 0.05, ** P < 0.01 versus Veh + LPS

Amyloidogenesis represents one of the keys in the pathogenesis of AD. To investigate whether the hippocampal GPR17 influenced amyloidogenesis inhibition in the mouse brain, we performed Western blotting. As shown in Fig. 4A–C and H–J, LV-GPR17–shRNA and cangrelor pretreatment inhibited the expression of APP (LV-GPR17–shRNA: *F* [2, 9] = 29.29, *P* < 0.01, Fig. 4A, and B; cangrelor: *F* [3, 12] = 15.6, *P* < 0.01, Fig. 4H, and I), and BACE1 (LV-GPR17–shRNA: *F* [2, 9]  = 38.48, *P* < 0.01, Fig. 4A, and C; cangrelor: *F* [3, 12] = 32.03, *P* < 0.01, Fig. 4H, and J) in the hippocampus. In addition, LV-GPR17–shRNA and cangrelor pretreatment also significantly attenuated increased Aβ level in the hippocampus of LPS-treated mice (LV-GPR17–shRNA: *F *[2, 9] = 21.86, *P* < 0.01, Fig. 4D; cangrelor:* F *[3, 12] = 24.41, *P* < 0.01, Fig. 4K). Learning and memory impairments have been linked to AD, and cholinergic dysfunction is thought to contribute to the onset of AD [37]. Therefore, the effects of GPR17 on the balance of the cholinergic system were analyzed in mice. As expected, LV-GPR17–shRNA and cangrelor pretreatment restored the balance of the cholinergic system by suppressing AChE activity (LV-GPR17–shRNA: *F *[2, 9] = 22.1, *P* < 0.01, Fig. 4E; cangrelor:* F *[3, 12] = 19.66, *P* < 0.01, Fig. 4L) and elevating ChAT activity (LV-GPR17–shRNA: *F *[2, 9] = 21.8, *P* < 0.01, Fig. 4F; cangrelor:* F *[3, 12] = 27.41, *P* < 0.01, Fig. 4M) as well as ACh level in the hippocampus (LV-GPR17–shRNA: *F *[2, 9]  = 15.85, *P* < 0.01, Fig. 4G; cangrelor:* F *[3, 12] = 10.56, *P* < 0.01, Fig. 4N).

To explore the antioxidant potential of LV-GPR17–shRNA and cangrelor against LPS-induced oxidative stress, we measured the levels of malondialdehyde (MDA) and superoxide dismutase (SOD) in the hippocampus. SOD is the main antioxidant enzyme in tissues, and MDA is the marker of lipid peroxidation. As shown in Fig. 4O–R, the MDA level in the hippocampus of LPS-treated mice was significantly higher than the control group (LV-GPR17–shRNA: *F *[2, 9] = 28.72, *P* < 0.01, Fig. 4O; cangrelor:* F *[3, 12] = 27.6, *P* < 0.01, Fig. 4Q). However, the SOD level in LPS-treated mice was greatly reduced compared to the control group both in the hippocampus (LV-GPR17–shRNA: *F *[2, 9] = 32.26, *P* < 0.01, Fig. 4P; cangrelor:* F *[3, 12] = 35.74, *P* < 0.01, Fig. 4R). LV-GPR17–shRNA and cangrelor pretreatment could decrease the level of MDA compared to the LPS-treated group and could increase the level of SOD in the hippocampus. Taken together, the above results suggest that the knockdown and inhibition of hippocampal GPR17 inhibits the production of amyloidogenesis and oxidative response in LPS-treated mice.

### Knockdown and inhibition of hippocampal GPR17 inhibits neuroinflammation induced by LPS in mice

**Fig. 5:**
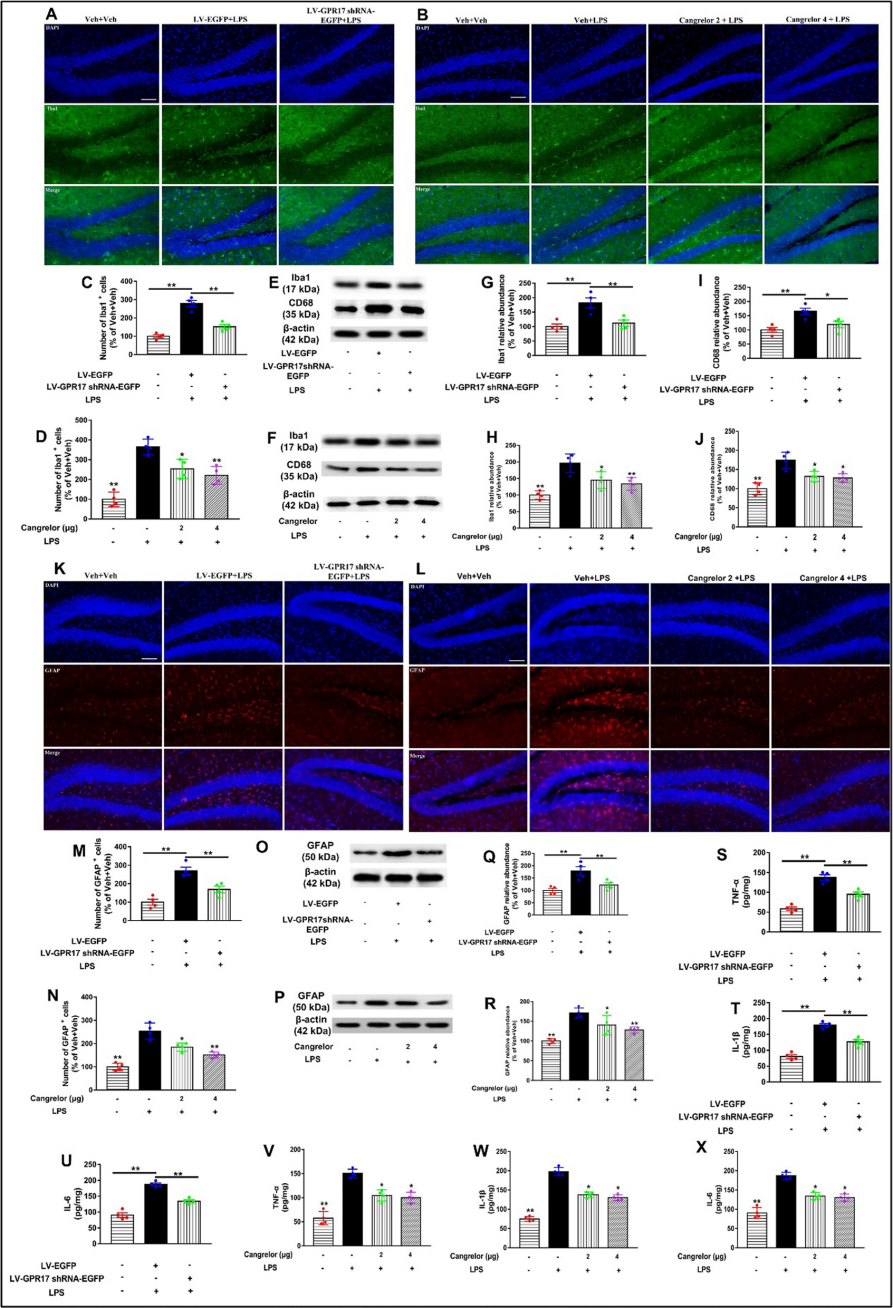
Knockdown and inhibition of hippocampal GPR17 inhibits neuroinflammation induced by LPS in mice. Representative images of immunofluorescent staining of Iba1 (green) and DAPI (blue) in the hippocampal DG of lentivirus (**A**) and cangrelor (**B**) pretreatment mice. Scale bars = 100 μm. The number of Iba1 antibody-stained microglia, in the DG of LV-GPR17–shRNA (**C**) and cangrelor (**D**) pretreatment mice. Iba-1 and CD68 protein levels were detected by Western blotting in the hippocampus of lentivirus (**E**) and cangrelor (**F**) pretreatment mice. Protein band intensity was normalized to β-actin. **G**–**J** Quantification of Iba-1 and CD68 protein levels is expressed as the fold difference relative to the control group. Representative images of GFAP (red) immunofluorescent staining in the hippocampal DG of the LV-GPR17–shRNA (**K**) and cangrelor (**L**) pretreatment groups. Scale bars = 100 μm. **M**, **N** Number of GFAP antibody-stained astrocytes was normalized, as the ratio (in percentage) of the Veh + Veh is shown. GFAP protein levels were detected by Western blotting in the mice of pretreatment with LV-GPR17–shRNA (**O**) and cangrelor (**P**). Protein band intensity was normalized to β-actin. **Q**, **R** Quantification of GFAP protein levels is expressed as the fold difference relative to the control group. **S**–**U** Pro-inflammatory cytokine TNF-α, IL-1β, and IL-6 in the brain were measured by ELISA in the mice of pretreatment with LV-GPR17–shRNA. **V**–**X** Pro-inflammatory cytokine TNF-α, IL-1β, and IL-6 in the brain were measured by ELISA in the mice of pretreatment with cangrelor. Data shown are expressed as mean ± SEM; *n* = 4 mice/group. *P < 0.05, **P < 0.01 versus Veh + LPS

Neuroinflammation mediated by glial activation and inflammatory cytokine release plays an important role in the progression of neurodegenerative diseases [38]. Neuroinflammation, characterized by overactivation of glial cells, plays an important role in cognitive impairment. LPS is a powerful inflammation-inducing agent. To explore whether LPS-induced neuroinflammation in mice, the expression of Iba1 and GFAP, markers of microglia and astrocytes in the brain, through immunofluorescence and Western blot assay, respectively. The results showed that LPS caused obvious microglia activation in the mouse hippocampal DG region (LV-GPR17–shRNA: *F* [2, 9] = 41.2, *P* < 0.01, Fig. 5A, and C; Cangrelor: *F* [3, 12] = 26.45, *P* < 0.01, Fig. 5B, and D). Similarly, LPS also caused obvious astrocytes activation in the hippocampus (LV-GPR17–shRNA: *F* [2, 9] = 23.51, *P* < 0.01, Fig. 5K, and M; cangrelor: *F* [3, 12] = 24.9, *P* < 0.01, Fig. 5L, and N), which were reduced by LV-GPR17–shRNA and cangrelor pretreatment. Consistent with the immunofluorescence results, western blotting results also showed that LPS significantly upregulated the expression of microglia and astrocytes levels in the hippocampus of LPS-treated mice, and these increases were significantly downregulated by LV-GPR17–shRNA and cangrelor pretreatment (LV-GPR17–shRNA–EGFP: *F* [2, 9] = 11.61,* P* < 0.01 for the microglia cells, Fig. 5G; *F *[2, 9] = 10.36,* P* < 0.01 for the astrocytes cells, Fig. 5Q; cangrelor: *F* [3, 12] = 14.3,* P* < 0.01 for the microglia cells, Fig. 5H; *F* [3, 12] = 11.19,* P* < 0.01 for the astrocytes cells, Fig. 5R). We also detected the expression of glial cells in other regions of the hippocampus. The results showed that LPS also significantly activated microglia and astrocytes in the CA1 and CA3 regions of the hippocampus, but GPR17 knockdown had no significant effect on their expression (Additional file 1: Fig. S4). To confirm whether the enhanced density of microglia induced by LPS administration represents a population of activated microglia, we performed western blot analysis on the expression of CD68, a lysosomal protein that is often used as a marker for active phagocytosis of microglia cells. Strikingly, CD68 expression was also significantly increased in the hippocampus, following the LPS injection, despite increases in microglia numbers. However, LV-GPR17–shRNA–EGFP and cangrelor pretreatment significantly inhibited LPS-induced expression of CD68 in the hippocampus of mice (LV-GPR17–shRNA–EGFP: *F* [2, 9] = 10.28,* P* < 0.01, Fig. 5I; cangrelor: *F* [3, 12] = 15.08,* P* < 0.01, Fig. 5J). Nest, we also detected the levels of pro-inflammatory cytokines in the hippocampus by ELISA. One-way ANOVA revealed that the levels of TNF-α, IL-1β, and IL-6 were much higher in the LPS group compared with the control group, which was significantly suppressed by LV-GPR17–shRNA (LV-GPR17–shRNA–EGFP: *F* [2, 9] = 24.69,* P* < 0.01 for TNF-α, Fig. 5S; *F* [2, 9] = 23.67, *P* < 0.01 for IL-1β, Fig. 5T; *F* [2, 9] = 24.9,* P* < 0.01 for IL-6; Fig. 5U) and cangrelor pretreatment (Cangrelor: *F* [3, 12] = 23.41,* P* < 0.01 for TNF-α, Fig. 5V; *F* [3, 12] = 30.59, *P* < 0.01 for IL-1β, Fig. 5W; *F* [3, 12] = 25.33,* P* < 0.01 for IL-6, Fig. 5X). Our results suggested that knockdown and inhibition of hippocampal GPR17 ameliorate the learning and memory ability by reducing LPS-induced neuroinflammation in mice.

### Knockdown and inhibition of hippocampal GPR17 ameliorated synaptic impairment and neuron apoptosis in LPS-treated mice

**Fig. 6:**
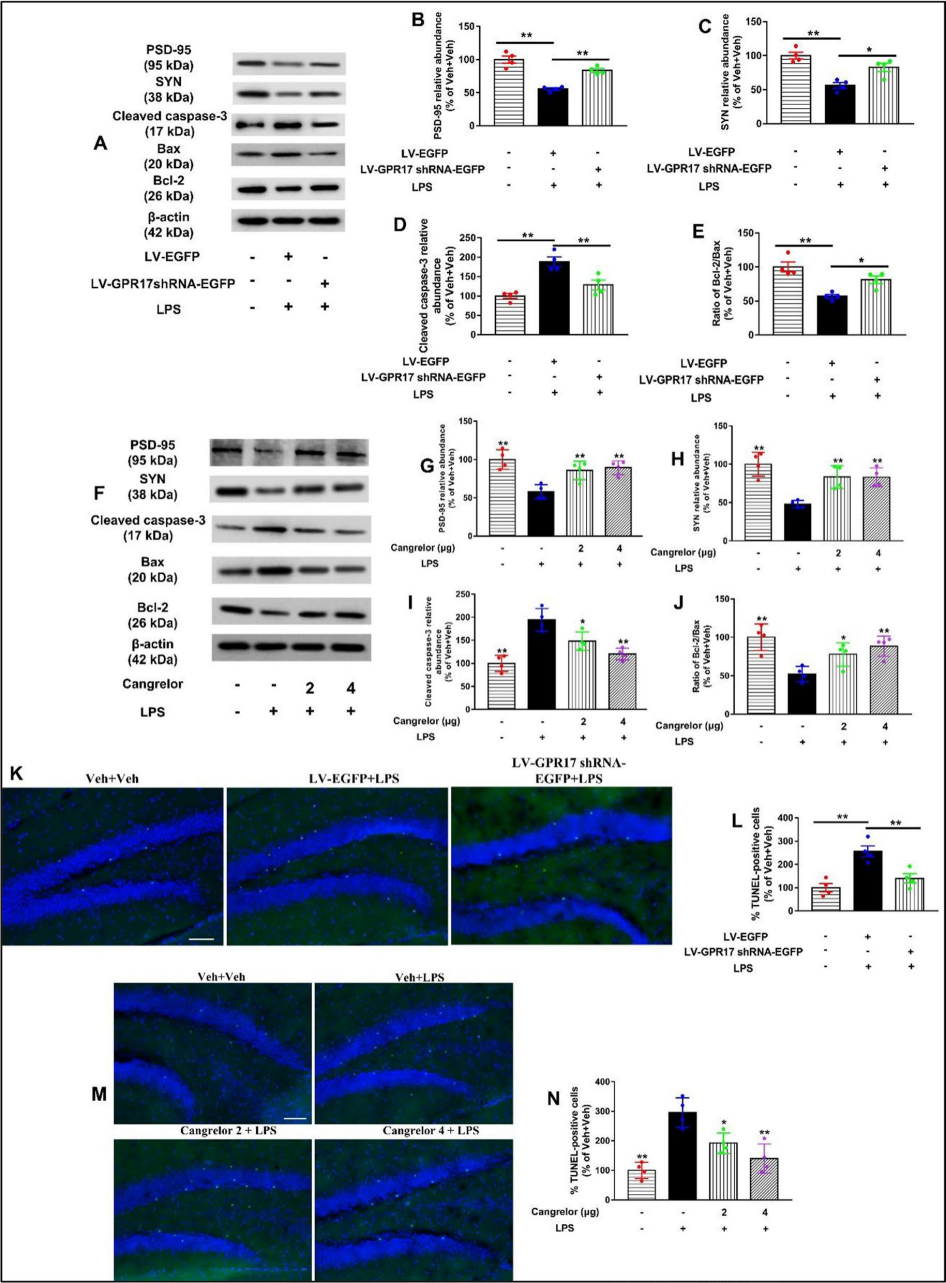
Knockdown and inhibition of hippocampal GPR17 ameliorated synaptic impairment and neuron apoptosis in LPS-treated mice. **A** Representative bands of PSD-95, SYN, cleaved caspase-3, Bcl-2, and Bax in the hippocampus of lentivirus groups were shown. Quantification of PSD-95 (**B**), SYN (**C**), cleaved caspase-3 (**D**), and (**E**) Bcl-2/Bax protein levels are expressed as the fold difference relative to the control group. **F** Representative bands of PSD-95, SYN, cleaved caspase-3, Bcl-2, and Bax in the hippocampus of cangrelor groups were shown. Quantification of PSD-95 (**G**), SYN (**H**), cleaved caspase-3(I), (**J**) Bcl-2/Bax protein levels is expressed as the fold difference relative to the control group. Protein band intensity was normalized to β-actin. Representative photographs of TUNEL-positive cells in the hippocampal DG of the LV-GPR17–shRNA (**K**) and cangrelor (**M**) groups. **L**, **N** Quantitative analysis of TUNEL-positive cells. Values shown are expressed as mean ± SEM; *n* = 4 mice/group. *P < 0.05, **P < 0.01 versus Veh + LPS. Scale bar, 100 μm

LPS-induced decrease in synaptic protein levels may contribute to the impairment of synaptic plasticity and the learning and memory decline observed in behavioral tests, suggesting impairments of synaptic plasticity may be responsible for LPS-induced memory deficits. The synapse-associated proteins, especially postsynaptic PSD-95 and presynaptic SYN promote synaptic plasticity. In this study, the levels of presynaptic PSD-95 (LV-GPR17–shRNA: *F* [2, 9] = 17.82, *P* < 0.01, Fig. 6A, and B; cangrelor: *F* [3, 12] = 12.13, *P* < 0.01, Fig. 6F, and G) and SYN (LV-GPR17–shRNA: *F* [2, 9] = 10.86, *P* < 0.01, Fig. 6A, and C; cangrelor: *F* [3, 12] = 7.45, *P* < 0.01, Fig. 6F, and H) decreased in the hippocampus of mice after the mice were treated with LPS. Meaningfully, in our study, knockdown and pharmacological blockade of GPR17 prevented the LPS-induced reduction of PSD-95 and SYN in the hippocampus of mice. These results suggested that knockdown and pharmacological blockade of GPR17 protected against the reduction of synaptic-associated protein in LPS-treated mice.

The anti-apoptotic effects of LV-GPR17–shRNA and cangrelor were investigated by detecting apoptotic-related proteins in the hippocampus. The results showed that the expression of active caspase-3 was induced by LPS but was decreased by LV-GPR17–shRNA and cangrelor pretreatment (LV-GPR17–shRNA: *F* [2, 9] = 17.29, *P* < 0.01, Fig. 6A, D; cangrelor: *F* [3, 12] = 18.39, *P* < 0.01, Fig. 6F, I). To assess whether the balance of hippocampal proapoptotic protein Bax and anti-apoptotic protein Bcl-2 was affected by LPS, the expression of Bax and Bcl-2 proteins was measured using western blot analysis. Our results showed that the expression of Bcl-2 protein was significantly decreased in the LPS-treated mice, while LV-GPR17–shRNA and cangrelor pretreatment were able to restore its expression comparable to the Veh + Veh group. In addition, the Bax expression was significantly less than that of Veh + LPS group, and the ratio of Bcl-2/Bax was markedly increased by LV-GPR17–shRNA and cangrelor pretreatment (LV-GPR17–shRNA: *F* [2, 9] = 15.2, *P* < 0.01, Fig. 6A, E; cangrelor: *F* [3, 12] = 8.12, *P* < 0.01, Fig. 6F, J).

To further observe the effects of LV-GPR17–shRNA and cangrelor on LPS-induced hippocampal neural cells, a TUNEL assay was performed analysis. We found that LPS injection increased TUNEL-positive cells (apoptosis) as compared with the control group in the hippocampal DG region (LV-GPR17–shRNA: *F* [2, 9] = 15.52, *P* < 0.01, Fig. 6K, L; cangrelor: *F* [3, 12] = 16.76, *P* < 0.01, Fig. 6M, N). Consistent with the above findings, LV-GPR17–shRNA and cangrelor pretreatment significantly attenuated LPS-induced neural cell apoptosis in the hippocampal DG region. We further assessed the cell death by co-localizing TUNEL-positive nuclei with the cell-specific marker NeuN. As demonstrated in Additional file 1: Fig. S5, TUNEL-positive nuclei were co-localized with NeuN. Pretreatment with LV-GPR17–shRNA and cangrelor significantly attenuated the LPS-induced increase of TUNEL^+^–NeUN^+^-positive cells in the hippocampal DG region (LV-GPR17–shRNA: *F* [2, 9] = 15.80, *P* < 0.01, Fig. S5A, C; cangrelor: *F* [3, 12] = 10.96, *P* < 0.01, Additional file 1: Fig. S5B, D). These data suggested that knockdown and inhibition of hippocampal GPR17 might ameliorate the learning and memory ability by reducing LPS-induced hippocampal neuron apoptosis in mice.

### Knockdown and inhibition of hippocampal GPR17 suppresses LPS-activated NF-κB–CREB/BDNF signaling pathway in mice

**Fig. 7:**
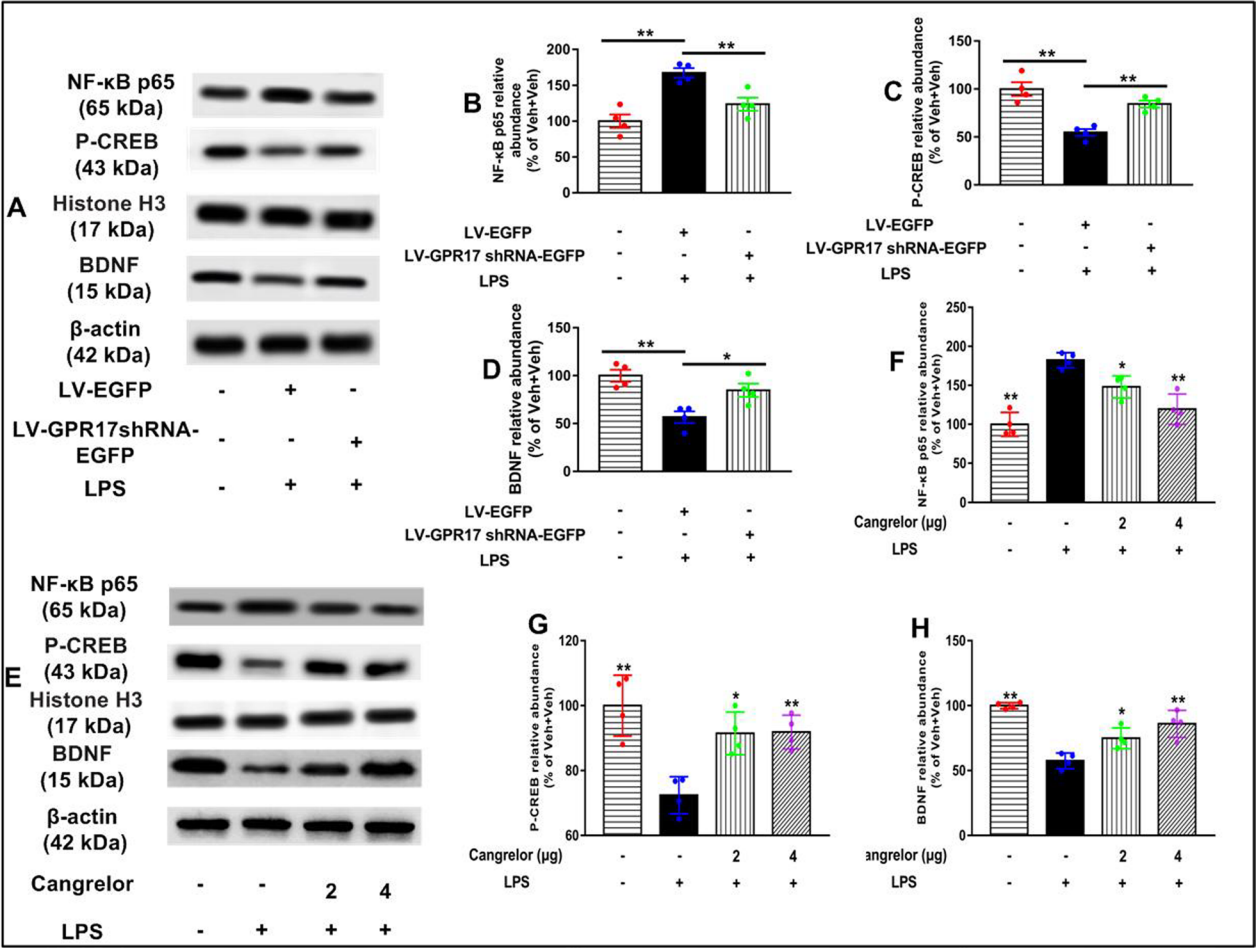
Knockdown and inhibition of hippocampal GPR17 suppresses LPS-activated NF-κB-CREB/BDNF signaling pathway in mice. **A** Representative bands of NF-κB p65, p-CREB, and BDNF in the hippocampus of lentivirus groups. Quantification of NF-κB p65 (**B**), p-CREB (**C**), and BDNF (**D**) protein levels is expressed as the fold difference relative to the control group. (**E**) Representative bands of NF-κB p65, p-CREB, and BDNF in the hippocampus of cangrelor groups. Quantification of NF-κB p65 (**F**), p-CREB (**G**), and BDNF (**H**) protein levels in the hippocampus. The band densities were normalized to the band density of the loading control actin. The extraction process of nuclear protein in mouse hippocampus as described above. Data shown are expressed as mean ± SEM; *n* = 4 mice/group. *P < 0.05, ** P < 0.01 versus Veh + LPS

GPR17 has been reported to be implicated in neuroinflammation by regulating the NF-κB signaling pathway, and LPS exposure activates NF-κB signaling by inducing the nuclear translocation of p65. To explore whether GPR17 is involved in LPS-activated NF-κB signaling, the hippocampal level of NF-κB p65 in the nuclear fraction was detected by Western blot analysis. As NF-κB p65 is a crucial transcription factor for the regulation of a wide range of genes, including the pro-inflammatory cytokines, then, we checked the nuclear expression of NF-κB p65 in the hippocampus. The level of NF-κB p65 was significantly higher in the LPS group than that in the Veh + Veh group (LV-GPR17–shRNA: *F* [2, 9] = 16.21, *P* < 0.01, Fig. 7A, and B; cangrelor: *F* [3, 12] = 8.12, *P* < 0.01, Fig. 7E, and F). However, shRNA-mediated knockdown and pharmacological blockade of GPR17 inhibited such nuclear translocation of NF-κB p65 (Fig. 7B, and F), suggesting inhibition of the nuclear translocation of NF-κB is a critical mechanism by GPR17 mediates its inhibitory effect on the pro-inflammatory cytokines. These results suggest that knockdown and pharmacological blockade of GPR17 inhibited LPS-induced neuroinflammation in the hippocampus via inhibition of NF-κB signaling. This data suggests that inhibition of the nuclear translocation of NF-κB p65 may be a critical mechanism by which LV-GPR17–shRNA and cangrelor mediate its protective effect against LPS-induced neuroinflammation and apoptosis in mice.

As shown in Fig. 7C, G, the levels of p-CREB in the hippocampus were decreased by LPS treatment (LV-GPR17–shRNA: *F* [2, 9] = 21.02, *P* < 0.01, Fig. 7A, C; cangrelor: *F* [3, 12] = 11.43, *P* < 0.01, Fig. 7E, G), which was reversed by LV-GPR17–shRNA and cangrelor pretreatment. The best-known transcriptional target of CREB is BDNF, which is known to emerge as an important synaptic modulator of synaptogenesis. BDNF and its signaling pathways are firmly implicated in neuronal differentiation and survival. Numerous pieces of evidence indicate that BDNF regulates both the early and late phases of long-term potentiation in the hippocampus. The levels of BDNF in the hippocampus were decreased in mice treated with LPS (LV-GPR17–shRNA: *F* [2, 9] = 11.62, *P* < 0.01, Fig. 7A, D; cangrelor: *F* [3, 12] = 23.92, *P* < 0.01, Fig. 7E, H), while it was dramatically recovered by LV-GPR17–shRNA and cangrelor pretreatment. These suggested that LV-GPR17–shRNA and cangrelor improve cognitive impairment induced by LPS and might be involved in the CREB/BDNF signaling in mice.

### Activation of hippocampal GPR17 induced cognitive impairment in normal mice

**Fig. 8:**
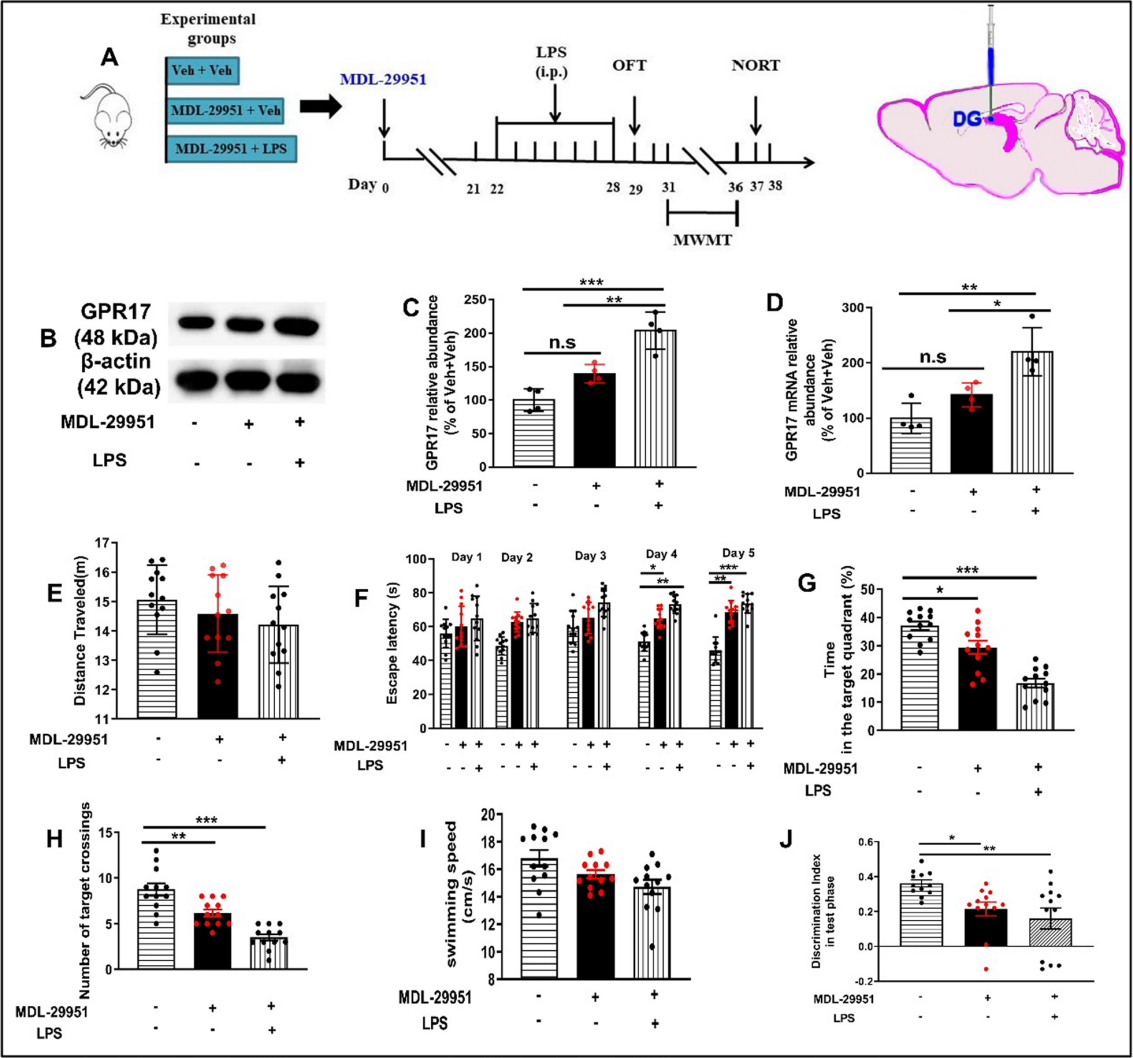
Activation of hippocampal GPR17 induced cognitive impairment in normal mice. **A** Experimental procedure for the test schedule. MDL-29951 was microinfused into bilateral DG regions of the hippocampus of mice, followed by LPS (0.25 mg/kg, i.p.) or its vehicle was administered 7 days after the MDL-29951 infusions 3 weeks, and then, the OFT, MWM, and NOR tests were conducted in mice. **B** Representative immunoreactive bands of GPR17 protein in the hippocampus. GPR17 protein (**C**) and mRNA levels (**D**) in the hippocampus, *n* = 4 mice/group. **E** Total distance in the OFT. **F** During the 2-day visible platform test, there were no differences in the escape latency among all groups in the MWM test. Escape latency was changed on the hidden platform during the 3-day acquisition trials. **G** Time spent in the target quadrant during the probe trial test. **H** Number of platform crossings during the probe trial test. **I** Swimming speed among all groups during probe testing on day 6. **J** Discrimination index of the NOR test. Values shown are expressed as mean ± SEM; *n* = 12 mice/group. *P < 0.05, **P < 0.01, ***P < 0.001

To verify the functional role of GPR17 in cognitive impairment from the negative side, we used its agonist MDL-29951 to further validate the functional role of GPR17 in learning and memory. Our results showed that MDL-29951 combined with LPS treatment increased the protein (*F* [3, 12] = 17.78, *P* < 0.01, Fig. 8B, and C), and the mRNA levels of GPR17 in the hippocampus of LPS-treated mice (*F* [3, 12] = 20.21, *P* < 0.01, Fig. 8D), but MDL-29951 had no significant effect on the expression of GPR17 protein and mRNA in the normal mice (*P* > 0.05, Fig. 8D).

To determine whether general locomotor activity interferes with cognitive behavior tests, we conducted the OFT. The results showed that there was no significant difference among the groups in the total distance traveled in the OFT (*F* [2, 33] = 0.97, *P* > 0.05, Fig. 8E). Then, we assessed the performance of mice in the visible-platform variant of the Morris water maze to test for baseline differences between treatment groups in vision and motivation. Mice in each group exhibited similar escape latency in the visible-platform test, suggesting no difference in vision or basal motivation among the three groups (Fig. 8F). We then tested the mice in the spatial hidden-platform variant. The results indicated that MDL-29951 only or MDL-29951 + LPS treatment, increased escape latencies compared to the control group (Fig. 8F). In the probe trial, the mice treated with MDL-29951 which displayed a significant decrease in the percentage of time (1 trial/mouse, *F* [2, 33] = 13.21*, P* < *0.05*, Fig. 8G) and the number of entries (1 trial/mouse, *F* [2, 33] = 21.57*, P* < *0.01*, Fig. 8H) in the target quadrant compared to the control. In contrast, mice treated with MDL-29951 plus LPS showed a significant decrease in both indices compared to the control (*P* < 0.001 for the percentage of time and* P* < 0.001 for the number of entries; Fig. 8G, H). In addition, all the mice showed similar swim distance and swim speed in the tests (*F* [2, 33] = 1.12, *P* > 0.05, Fig. 8I).

To confirm the results observed in the MWM test, we also carried out the NOR test. During the test period, mice treated with MDL-29951 caused a significant decline in the discrimination index compared with the control group (*F* [2, 33] = 17.25*, P* < 0.05, Fig. 8J). Mice treated with MDL-29951 plus LPS showed a significant decline in the discrimination index compared with the control group (*F* [2, 33] = 28.13*, P* < 0.01, Fig. 8J). These behavior data indicate that activation of hippocampal GPR17 induced cognitive impairment in normal mice.

### Mechanisms of cangrelor against LPS-induced neuroinflammation in BV-2 cells

**Fig. 9:**
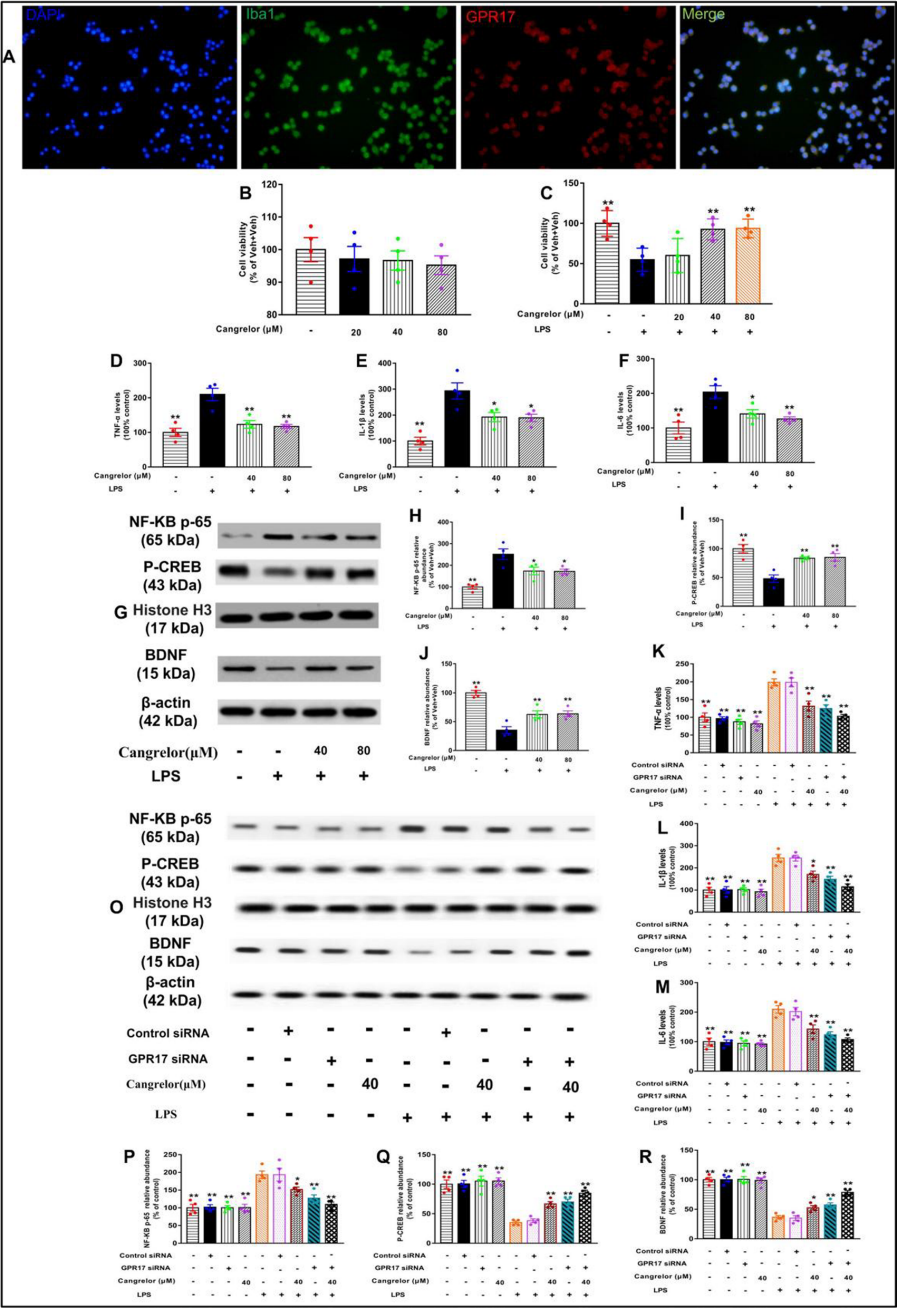
Mechanism of cangrelor against LPS-induced neuroinflammation in BV-2 cells. **A** Immunofluorescence staining of GPR17 (red), Iba1 (green), and DAPI (blue) in the BV-2 cells. BV-2 cell viability was examined by CCK8 assay after different concentrations of cangrelor (20, 40, and 80 μM) for 24 h (**B**) and after LPS (1 µg/ml, 24 h) stimulation with or without cangrelor (**C**). The levels of TNF-α (**D**), IL-1β (**E**), and IL-6 (**F**) in BV-2 cells treated with LPS (1.0 µg/ml) for 24 h with or without cangrelor pretreatment. The expressions of NF-κB p65, p-CREB, and BDNF were examined by Western blotting in BV-2 cells treated with LPS (1 µg/ml) for 24 h with or without cangrelor pretreatment (**G**). Quantification of NF-κB p65 (**H**), p-CREB (**I**), and BDNF (**J**) protein levels are expressed as the fold difference relative to the control group. After incubation with GPR17 siRNA or its control siRNA for 48 h, BV-2 cells were administrated with 40 μM cangrelor for 1 h followed by LPS (1.0 µg/ml) for an additional 24 h. The levels of TNF-α (**K**), IL-1β (**L**) and IL-6 (**M**). The expressions of NF-κB p65, p-CREB, and BDNF were examined by western blotting (**O**). Quantification of NF-κB p65 (**P**), p-CREB (**Q**), and BDNF (**R**) was presented as the ratio (in percentage) of the Veh + Veh group. Data shown are expressed as mean ± SEM; *n* = 4. *P < 0.05, **P < 0.01 versus Veh + LPS

To determine the anti-neuroinflammatory activity of cangrelor, we first examined the drug toxicity of cangrelor in BV-2 microglia cells. BV-2 cells were treated with cangrelor (20, 40, and 80 μM) for 24 h, and cell viability was determined. Our results showed that cangrelor did not exhibit toxicity at different doses, thus excluding the possibility of toxic effects of cangrelor (*P* > 0.05, Fig. 9B). Subsequently, we examined the inhibitory effect of cangrelor on microglial activation. To investigate the potential regulatory effects of cangrelor on pro-inflammatory responses, BV-2 cells were treated with vehicle or cangrelor (20, 40, and 80 μM) for 30 min, followed by LPS (1 μg/ml) or PBS. Our results showed that cangrelor exerted potent neuroprotective effects following LPS insult by maintaining cell viability (*F* [4, 15] = 7.31, *P* < 0.01, Fig. 9C). We also determined that 40 and 80 μM of cangrelor were the optimum dose for neuroprotective activity. To determine the efficacy of cangrelor in modulating neuroinflammatory responses at the cellular level, we examined the levels of inflammatory cytokines. After LPS stimulation, the levels of the pro-inflammatory cytokines including TNF-α, IL-1β, and IL-6 were significantly increased, while pretreatment with 40 and 80 μM of cangrelor could significantly inhibit the levels of TNF-α, IL-1β, and IL-6 induced by LPS in BV-2 cells (TNF-α: *F* [3, 12] = 15.82, *P* < 0.01, Fig. 9D; IL-1β: *F* [3, 12] = 14.81, *P* < 0.01, Fig. 9E; IL-6: *F* [3, 12] = 9.41, *P* < 0.01, Fig. 9F). In addition, cangrelor significantly down-regulated the expression of NF-κB p65 and up-regulated the expression of p-CREB and BDNF proteins in LPS-treated BV-2 cells (NF-κB p65: *F* [3, 12] = 14.03, *P* < 0.01, Fig. 9G, and H; p-CREB: *F* [3, 12] = 13.95, *P* < 0.01, Fig. 9G, and I; BDNF: *F* [3, 12] = 24.79, *P* < 0.01, Fig. 9G, and J). To further acknowledge the role of GPR17 in cangrelor against LPS-elicited neuroinflammation, GPR17 expression was knocked down using siRNA. The results revealed that pretreatment with cangrelor or GPR17 siRNA inhibited the levels of pro-inflammatory cytokines (TNF-α: *F* [8, 27] = 20.31, *P* < 0.01, Fig. 9K; IL-1β: *F* [8, 27] = 20.62, *P* < 0.01, Fig. 9L; IL-6: *F* [8, 27] = 19.02, *P* < 0.01, Fig. 9M) and NF-κB p65, and increased the expression of p-CREB and BDNF in LPS-treated BV-2 cells (NF-κB p65: *F* [8, 27] = 15.84, *P* < 0.01, Fig. 9O, and P; p-CREB: *F* [8, 27] = 25.95, *P* < 0.01, Fig. 9O, and Q; BDNF: *F* [8, 27] = 23.58, *P* < 0.01, Fig. 9O, and R). Moreover, the combination of cangrelor or GPR17 siRNA significantly inhibited the expression of NF-κB p65 and pro-inflammatory cytokines and increased the expression of p-CREB and BDNF, which suggested that GPR17-mediated signaling might be critical in ensuring the beneficial effects of cangrelor on neuroinflammation.

In addition, we examined the effects of MDL-29951 on inflammatory cytokines. The levels of TNF-α, IL-1β, and IL-6 in the supernatant of the culture medium were determined by the ELISA method. The results showed that MDL-29951 treatment significantly increased the levels of TNF-α, IL-1β, and IL-6 in BV-2 cells, compared with the control group (Additional file 1: Fig. S6).

## Discussion

The purpose of this study was to identify the role of GPR17 on LPS-induced cognitive deficit. Increasing evidence indicates that neuroinflammation may play a crucial role in the development of cognitive deficits [39, 40]. LPS has been used to study neuroinflammation-associated behavioral and pathological changes in the brain [41]. Studies have reported increased expression of GPR17 in patients with traumatic brain injury, cerebral ischemia, and other animal models of central nervous system damage [27, 42]. In addition, we also found that GPR17 expression was significantly upregulated in the hippocampus of Aβ_1–42_-induced mouse models of cognitive impairment. Whether LPS exposure can also cause GPR17 expression changes in the brain? With that in mind, we have conducted a number of experiments. Interestingly, our result found that GPR17 expression in the hippocampus of mice was also increased after LPS treatment. However, knockdown and inhibition of GPR17 prevented LPS-induced cognitive deficits. In addition, we also found that activation of hippocampal GPR17 with MDL-29951 induced cognitive impairment in mice. Pharmacological activation of GPR17 leads to an increase in the release of pro-inflammatory cytokines, including TNF-α, IL-1β, and IL-6 on BV-2 cells. These results suggest that GPR17 may be involved in the regulation of the pathogenesis of cognitive impairment, but the underlying molecular mechanisms remain unclear. In addition, why did cangrelor (GRP17 inhibitor) reduce GPR17 expression in the mice? We think it may involve the negative feedback regulation. The mechanism of cangrelor inhibiting LPS-induced up-regulation of GPR17 expression needs to be further studied. GPR17 can be activated by both the uracil nucleotide (UDP, and UDP glucose, UDP galactose) and the cysteamine aminoacyl leukotriene (LTC4 and LTD4) agonist families [43, 44]. However, activation of the GPR17 receptor by cysteinyl-leukotriene and purinergic ligands is still controversially discussed. In fact, some researchers could not reproduce GPR17 activation by either ligand type in various assay systems. Whether the expression level of the GPR17 ligand is increased due to LPS treatment is also unknown and needs further study.

Previous study demonstrated that repeated administration of LPS causes inflammation and amyloidogenesis due to increased Aβ formation, and lead to cognitive degradation during the process of AD [5, 45]. It was also reported that the injection of LPS led to the accumulation of Aβ through β- and γ-secretase activities [46]. Similar to previous results, our study demonstrated this effect, with the LPS-treated mice displaying marked increases in Aβ content in the brain. This was associated with enhanced BACE1 activity, a limiting enzyme that produces Aβ and is known to be induced by NF-κB and inflammatory cytokines, as observed here. GPR17 has been reported to regulate the NF-κB signaling pathway [30, 31]. Interestingly, knockdown and inhibition of hippocampal GPR17 succeeded in preventing the LPS-induced increase in the Aβ content, which may be related to the reduction in BACE1 activity. Herein, we found that knockdown and inhibition of hippocampal GPR17 significantly reduced the expression of APP and BACE1 proteins in the mouse hippocampus. Mounting evidence indicates that AChE plays a critical role in learning and memory [8, 47]. Altered AChE activity is associated with cognitive decline observed in the neuroinflammatory response [48]. It has been shown that AChE activity increases with exposure to LPS [37]. Similar to previous reports, our results showed elevated AChE activity in the hippocampus of mice induced by LPS, which were reversed by LV-GPR17–shRNA and cangrelor pretreatment. As a critical neurotransmitter in the brain, ACh is synthesized by ChAT but hydrolyzed by AChE. As expected, LPS was found to cause a decrease in Ach levels by inhibiting ChAT activity and increasing AChE activity. However, knockdown and inhibition of hippocampal GPR17 markedly normalized the cholinergic system in the mouse brain.

Some studies have recently shown that LPS activates neuronal and glial cells, which secrete neurotoxic factors, and cause neuroinflammation in the brain. Microglia are the innate immune cells in the brain that play an important role in neuroinflammation [49, 50]. Astrocytes are a major class of glial cells that respond to neuronal activity via Ca^2+^ intracellular transients, and then release transmitters that alter synaptic connectivity [51]. Astrocytes are also active participants in propagating and regulating neuroinflammation [52]. Thus, inhibition of activated microglia and astrocytes cells can help to ameliorate the severity of neuroinflammation. GPR17 was reported to be found in both neurons and glial cells [42, 53]. GPR17 mediates microglial inflammation in the chronic phase of cerebral ischemia and regulates allergic pulmonary inflammation [25]. Meanwhile, a previous study verified that GPR17 knockdown attenuated the chronic injury and microgliosis as indicated by a reduction in Iba1-positive microglia [27]. Cangrelor decreased the inflammatory response and inhibited the expression of inflammatory cytokines [54]. Therefore, we analyzed the expression of Iba1 and GFAP, activation markers of microglia and astrocytes, respectively, through immunofluorescence and western blot analyses, and found that LV-GPR17–shRNA and cangrelor pretreatment significantly inhibits their overactivation by LPS injection. Here, we also observed an increase in the expression of CD68, which could be interpreted as a marker of pro-inflammatory activation in the context of LPS exposure. However, LV-GPR17–shRNA and cangrelor pretreatment also significantly inhibit the expression of CD68 in the mouse hippocampus.

NF-κB can be activated by LPS and induces several inflammatory [55, 56]. NF-κB also induces the decomposition of APP by activating BACE1 [57]. NF-κB is activated by inflammatory intermediates and oxidative stress. The expression of several inflammatory genes such as inflammatory cytokines could be regulated by NF-κB activation [58]. The “cholinergic anti-inflammatory pathway” is mediated by ACh-binding to the α7 nicotinic receptor, which inhibits NF-κB activation and the production of pro-inflammatory cytokines [59]. Furthermore, NF-κB signaling enhances apoptosis, and inhibition of it not only protracts inflammatory responses but also prevents apoptosis [18]. GPR17 has been found to regulate NF-κB activity [30]. We speculate that the anti-inflammation and anti-apoptosis effects of LV-GPR17–shRNA and cangrelor might be mediated through the NF-κB signaling pathway. Oxidative stress is also known to damage cognitive function and is considered to have a role in the etiology of neurodegenerative disorders, according to evidence [60]. Indeed, neuroinflammation and oxidative stress coexist, and the interplay of oxygen-free radicals and inflammatory agents exacerbates cognitive deficits. The precise pathophysiology underpinning the effect of neuroinflammation and oxidative stress on cognitive performance, however, remains unknown. MDA is a key indicator of lipid peroxidation, which reflects the degree to which cells are attacked by free radicals. SOD is an essential antioxidant enzyme that catalyzes the disproportionation of superoxide anion to generate peroxides of oxygen and hydrogen, scavenging ROS and free radicals [61]. When the tissue was subjected to oxidative stress, SOD activity decreased and MDA level increased. In addition, a significant increase in LPS-induced oxidative stress has been reported, characterized by an increase in MDA levels and a decrease in SOD levels [9]. Our results showed that pretreatment with LV-GPR17–shRNA and cangrelor attenuated the LPS-induced increase in MDA and decrease in the activities of SOD in the hippocampus.

Previous research has demonstrated that cognitive and behavioral impairment is closely associated with neuronal loss in the hippocampus [62, 63]. LPS-induced neuroinflammation enhances neuronal apoptotic neurodegeneration [64]. Early cell changes that occur during apoptosis are mediated by the Bcl-2 family of proteins, including the anti-apoptotic Bcl-2 and pro-apoptotic Bax [19]. Caspase-3 is considered to be an important feature of apoptosis in neuronal cells. Activation of caspase-3 results in the death of neuronal cells [65]. GPR17 agonist profoundly increased reactive oxygen species in a time-dependent manner, thus leading to apoptosis [66]. Over-expression of GPR17 in the glioma cell lines U87MG and U251 induced apoptosis by increasing ROS levels [30]. In this study, these results are in agreement with previous findings. Our results showed that after exposure to LPS, the expression of the Bcl-2 protein decreased, while the expression of Bax increased. However, LV-GPR17–shRNA and cangrelor pretreatment downregulated the expression of Bax, upregulated the expression of Bcl-2, and mitigated caspase 3 activities and the number of apoptosis cells in LPS-treated mice. These findings suggest that knockdown and inhibition of hippocampal GPR17 may improve cognitive dysfunction by reducing LPS-induced neuronal apoptosis in the hippocampus.

Deficits in presynaptic SYN and postsynaptic PSD-95 correlate with cognitive decline in AD [67]. The accumulation of Aβ disrupts synaptic plasticity, further leading to neuron damage and memory loss [68]. Similar to previous studies, in this study, the levels of SYN and PSD-95 decreased in the hippocampus of LPS-exposed mice. This LPS-induced decrease in synaptic protein levels may contribute to the impairment of synaptic plasticity and the learning and memory decline observed in behavioral tests. In our study, knockdown and inhibition of hippocampal GPR17 prevented the LPS-induced reduction of SYN and PSD-95 in the hippocampus. Evidence suggests that BDNF plays a crucial role in neuronal plasticity, neuronal survival, and differentiation [69, 70]. It has been reported that LPS injection broadly reduces BDNF expression in the brain [9]. GPR17 may be involved in regulating the expression of BDNF [71]. In our research, we found decreased BDNF level in the hippocampus of LPS-treated mice, which was restored after LV-GPR17–shRNA and cangrelor pretreatment. The BDNF gene is controlled by several transcription factors, and CREB plays a central role in this regulatory process [72]. It has been shown that LPS reduced p-CREB expression in the brains of mice [73]. Our study shows a reduction in the expression of p-CREB in the hippocampus of mice administered with LPS but LV-GPR17–shRNA and cangrelor pretreatment were able to upregulate the expression of p-CREB in the hippocampus of the brain. Meanwhile, cangrelor could suppress the expressions of NF-κB P65, and inflammatory factors in LPS-treated BV-2 cells. The combination of cangrelor or GPR17 siRNA even lowered their expressions to near baseline levels, suggesting that GPR17-mediated signaling is involved in the protective effects of cangrelor on neuroinflammation. Moreover, cangrelor could increase the expression of p-CREB and BDNF in LPS-treated BV-2 cells, the combination of cangrelor or GPR17 siRNA even increased their expressions to near baseline levels. Therefore, these results suggest that knockdown and inhibition of GPR17 induced neuroprotection by inhibiting the LPS-induced activation of NF-κB inflammatory signaling and by enhancing the associated CREB/BDNF expression in LPS-treated mice.

## Conclusion

**Fig. 10:**
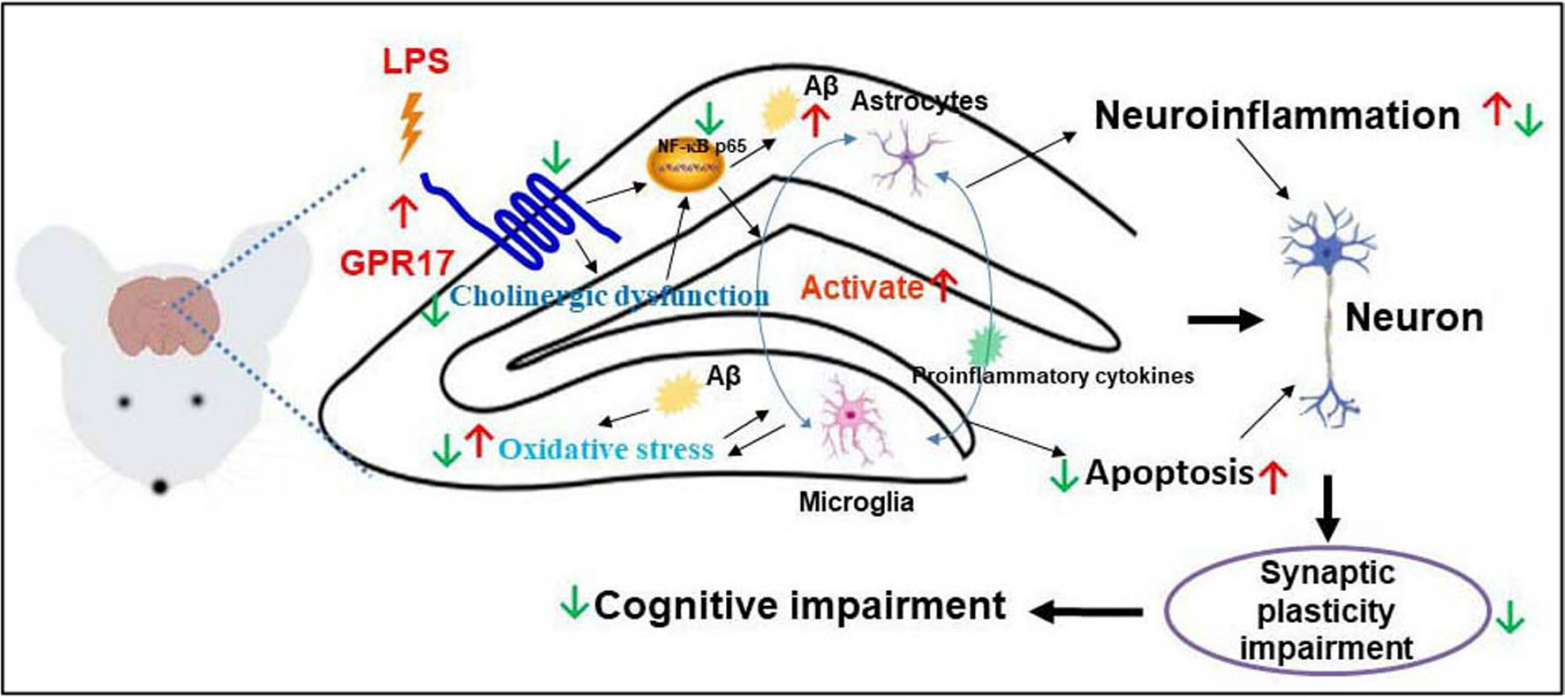
Knockdown and inhibition of hippocampal GPR17 attenuates LPS-induced cognitive impairment by inhibiting neuroinflammation, cholinergic dysfunction, oxidative stress, and synaptic damage in mice

In summary, our study indicates that knockdown and inhibition of hippocampal GPR17 display neuroprotective effects on LPS-induced memory impairment and neuroinflammation by modulation of NF-κB and CREB/BDNF signaling pathways in mice. The summary diagram is shown in Fig. 10. Our current research may provide a new drug target for the treatment of AD. However, there are still some limitations to this work. First, there is still an urgent need to design or find specific GPR17 inhibitors to detect their anti-AD effects. Second, the viruses can be engineered to intervene in specific types of cells. Finally, more comprehensive studies are required to support this notion and to better understand the exact protective mechanisms of GPR17 in the brain.

### Supplementary Information

**Additional file 1: Fig. S1.** The expression of EGFP from LV-GPR17-shRNA-EGFP in Iba1 antibody-stained cells in LPS-treated mice. Immunofluorescence staining of LV-GPR17-shRNA-EGFP (green), Iba1(red) in the hippocampal DG region of the LPS-treated mice. Scale bars = 100 μm.** Fig. S2.** GPR17 knockdown in normal mice had no effect on learning and memory.** A** Experimental procedure for the test schedule.** B** Representative immunoreactive bands of GPR17 protein in the hippocampus. β-actin was used as an internal control, and relative protein levels were quantified by densitometry analysis using Image J software. Quantification of GPR17 protein (**C**) and mRNA (**D**) levels in the hippocampus was shown,* n* = 4 mice/group.** E** The total distance in the OFT.** F** The escape latency among all groups in the MWM test.** G** The time spent in the target quadrant during the probe trial test.** H** The number of platform crossings during the probe trial test.** I** Swimming speed among all groups during probe testing on day 6.** J** The discrimination index of the NORT. Values shown are expressed as mean ± SEM;* n* = 12 mice/group. ** P < 0.01 versus Control group.** Fig. S3.** Pharmacological inhibition of hippocampus GPR17 in normal mice had no effect on learning and memory.** A** Experimental procedure for the test schedule.** B** Representative immunoreactive bands of GPR17 protein in the hippocampus. β-actin was used as an internal control, and relative protein levels were quantified by densitometry analysis using Image J software. Quantification of GPR17 protein (**C**) and mRNA (**D**) levels in the hippocampus was shown,* n* = 4 mice/group.** E** The total distance in the OFT.** F** The escape latency among all groups in the MWM test.** G** The time spent in the target quadrant during the probe trial test.** H** The number of platform crossings during the probe trial test.** I** Swimming speed among all groups during probe testing on day 6.** J** The discrimination index of the NORT. Values shown are expressed as mean ± SEM;* n* = 12 mice/group.** Fig. S4.** GPR17 knockdown had no significant effect on the expression of glial cells in the hippocampal CA1 and CA3 regions. Representative images of immunofluorescent staining of Iba1 (green) and DAPI (blue) in the hippocampal** A** CA1 and** B** CA3 region of lentivirus pretreatment mice. The number of Iba1 antibody-stained microglia, in the hippocampal CA1 and CA3 region of LV-GPR17-shRNA pretreatment mice. Representative images of immunofluorescent staining of GFAP (red) and DAPI (blue) in the hippocampal **C** CA1 and** D** CA3 region of lentivirus pretreatment mice. The number of GFAP antibody-stained astrocytes, in the hippocampal CA1 and CA3 region of lentivirus pretreatment mice.Values shown are expressed as mean ± SEM;* n* = 4 mice/group. **P < 0.01 versus Control group. Scale bars = 100 μm. **Fig. S5.** Knockdown and inhibition of hippocampal GPR17 ameliorated neuron apoptosis in LPS-treated mice.** A**,** B** TUNEL assay and DAPI nuclear stain were used to identify dead cell nuclei. TUNEL assay (green) was performed with immunofuorescence for NeuN (red), and DAPI (blue). TUNEL-positive nuclei were co-localized in NeuN-positive cells in the hippocampus.** C**,** D** Quantitative analysis of TUNEL+-NeuN+-positive cells. Values shown are expressed as mean ± SEM;* n* = 4 mice/group. *P < 0.05, **P <0.01 versus Veh+LPS. Scale bar, 100 μm.** Fig. S6.** MDL-29951 treatment increased inflammatory cytokines in BV-2 cells. The cells were pretreated with MDL-29951 (5,10, or 20 μM), Then, culture supernatant was collected to detect the production of TNF-α (**A**),** B** IL-1β, and IL-6 (**C**). Data shown are expressed as mean ± SEM;* n* = 4. *P < 0.05, **P <0.01, ***P <0.001 versus Control group.

## Data Availability

All data generated or analyzed during this study are included in this published article and its Additional file 1. Raw data that support the findings of this study are available from the corresponding author, upon reasonable request.

## Abbreviations

AD: Alzheimer’s disease
Aβ: Amyloid β
Ach: Acetylcholine
AChE: Acetylcholine esterase
BDNF: Brain-derived neurotrophic factor
ChAT: Choline acetyltransferase
CREB: CAMP response element binding protein
CNS: Central nervous system
DG: Dentate gyrus
GPR17: G protein-coupled receptor 17
ICR: Institute of cancer research
LPS: Lipopolysaccharide
MWM: Morris water maze
NOR: Novel object recognition
NF-κB: Nuclear factor kappa B
OFT: Open field test
PSD-95: Postsynaptic density protein 95
RT-PCR: Real-time quantitative PCR
SYN: Synaptophysin
