# Supplementary material for: Knockdown and inhibition of hippocampal GPR17 attenuates lipopolysaccharide-induced cognitive impairment in mice
Source: J Neuroinflammation. 2023 Nov 21;20:271. doi: 10.1186/s12974-023-02958-9 (PMC10662506; doi:10.1186/s12974-023-02958-9)
Supplement: Supplementary file 1 — Additional file 1: Fig. S1. The expression of EGFP from LV-GPR17-shRNA-EGFP in Iba1 antibody-stained cells in LPS-treated mice. Immunofluorescence staining of LV-GPR17-shRNA-EGFP (green), Iba1(red) in the hippocampal DG region of the LPS-treated mice. Scale bars = 100 μm. Fig. S2. GPR17 knockdown in normal mice had no effect on learning and memory. A Experimental procedure for the test schedule. B Representative immunoreactive bands of GPR17 protein in the hippocampus. β-actin was used as an internal control, and relative protein levels were quantified by densitometry analysis using Image J software. Quantification of GPR17 protein (C) and mRNA (D) levels in the hippocampus was shown, n = 4 mice/group. E The total distance in the OFT. F The escape latency among all groups in the MWM test. G The time spent in the target quadrant during the probe trial test. H The number of platform crossings during the probe trial test. I Swimming speed among all groups during probe testing on day 6. J The discrimination index of the NORT. Values shown are expressed as mean ± SEM; n = 12 mice/group. ** P < 0.01 versus Control group. Fig. S3. Pharmacological inhibition of hippocampus GPR17 in normal mice had no effect on learning and memory. A Experimental procedure for the test schedule. B Representative immunoreactive bands of GPR17 protein in the hippocampus. β-actin was used as an internal control, and relative protein levels were quantified by densitometry analysis using Image J software. Quantification of GPR17 protein (C) and mRNA (D) levels in the hippocampus was shown, n = 4 mice/group. E The total distance in the OFT. F The escape latency among all groups in the MWM test. G The time spent in the target quadrant during the probe trial test. H The number of platform crossings during the probe trial test. I Swimming speed among all groups during probe testing on day 6. J The discrimination index of the NORT. Values shown are expressed as mean ± SEM; n = 12 mice/group. Fig. S4. [file 12974_2023_2958_MOESM1_ESM.docx]

**Additional materials**

**Knockdown and inhibition of hippocampal GPR17** **attenuates** **lipopolysaccharide-induced cognitive impairment in mice**

**figures and legends**

**
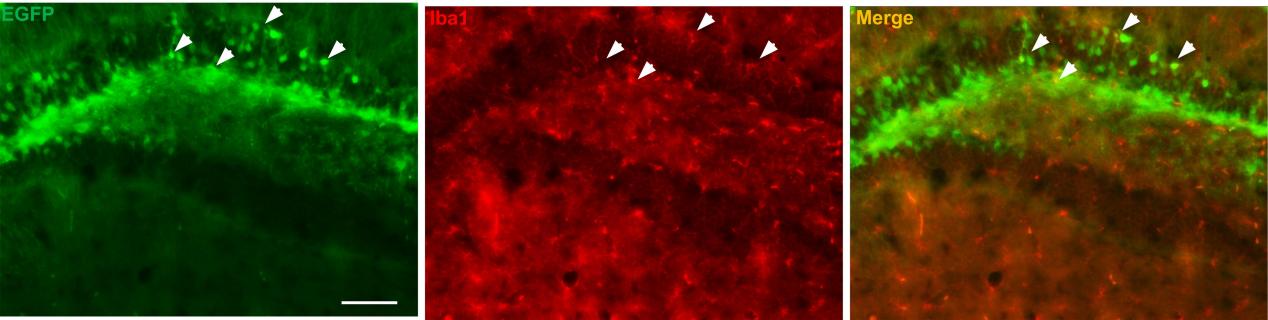
**

**Fig. S1. The expression of EGFP from LV-GPR17-shRNA-EGFP in Iba1 antibody-stained cells in LPS-treated mice.** Immunofluorescence staining of LV-GPR17-shRNA-EGFP (green), Iba1(red) in the hippocampal DG region of the LPS-treated mice. Scale bars = 100 μm.


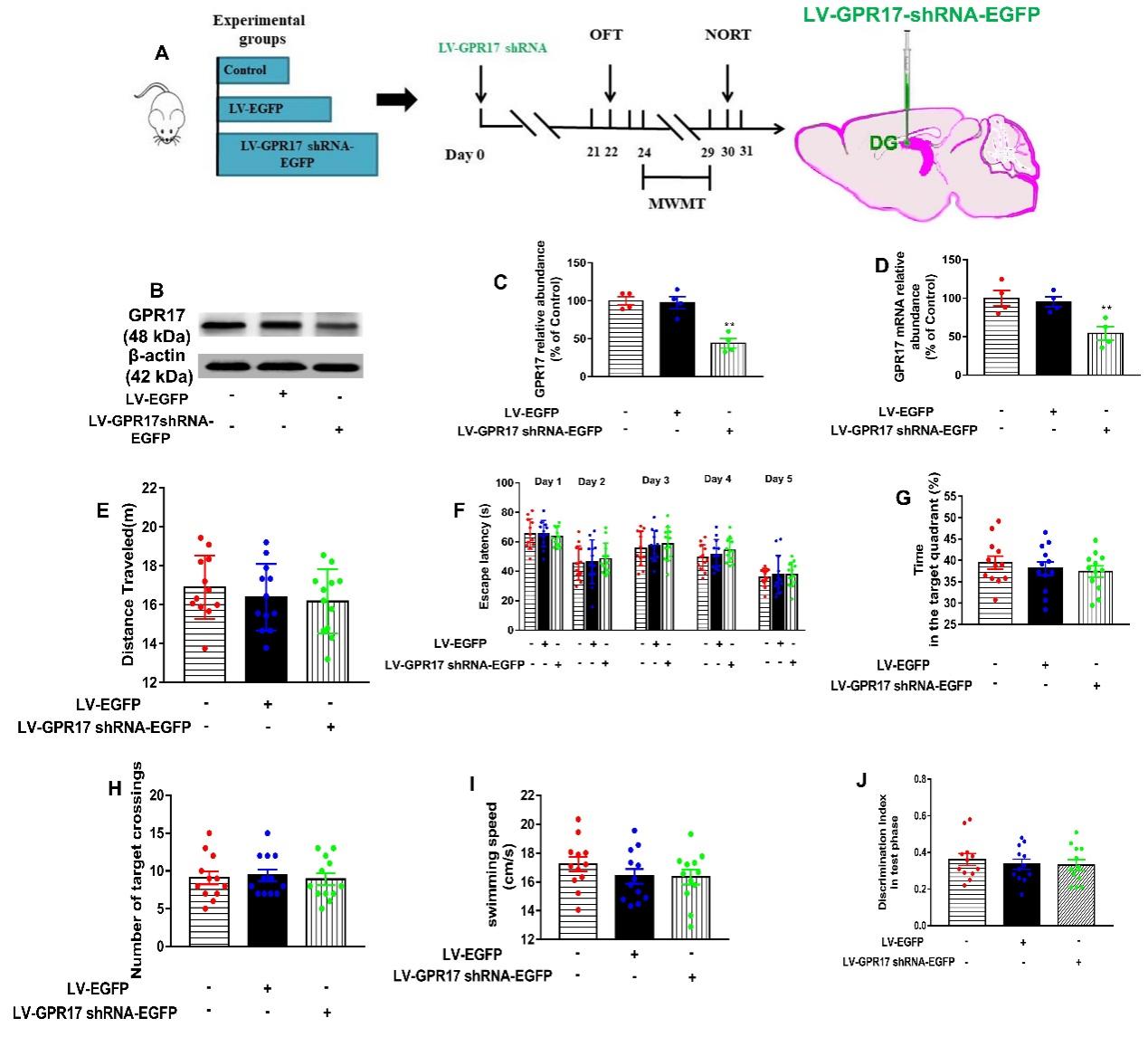


**Fig. S2.** **GPR17 knockdown in normal mice had no effect on learning and memory.** (A) Experimental procedure for the test schedule. (B) Representative immunoreactive bands of GPR17 protein in the hippocampus. β-actin was used as an internal control, and relative protein levels were quantified by densitometry analysis using Image J software. Quantification of GPR17 protein (C) and mRNA (D) levels in the hippocampus was shown, n=4 mice/group. (E) The total distance in the OFT. (F) The escape latency among all groups in the MWM test. (G) The time spent in the target quadrant during the probe trial test. (H) The number of platform crossings during the probe trial test. (I) Swimming speed among all groups during probe testing on day 6. (J) The discrimination index of the NORT. Values shown are expressed as mean ± SEM; n=12 mice/group. *** P <0.01* versus Control group.


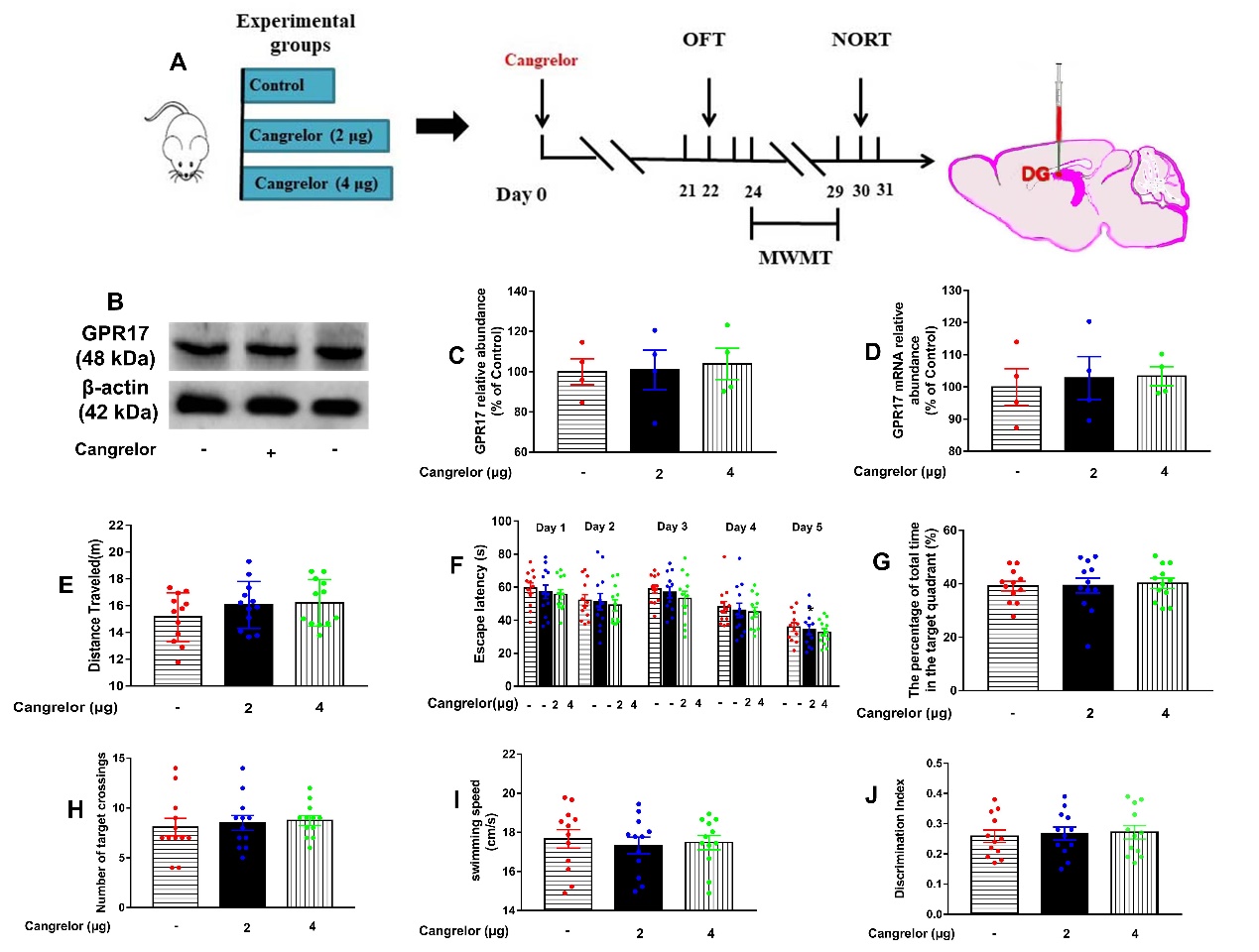


**Fig. S3.** **Pharmacological inhibition of hippocampus GPR17 in normal mice had no effect on learning and memory.** (A) Experimental procedure for the test schedule. (B) Representative immunoreactive bands of GPR17 protein in the hippocampus. β-actin was used as an internal control, and relative protein levels were quantified by densitometry analysis using Image J software. Quantification of GPR17 protein (C) and mRNA (D) levels in the hippocampus was shown, n=4 mice/group. (E) The total distance in the OFT. (F) The escape latency among all groups in the MWM test. (G) The time spent in the target quadrant during the probe trial test. (H) The number of platform crossings during the probe trial test. (I) Swimming speed among all groups during probe testing on day 6. (J) The discrimination index of the NORT. Values shown are expressed as mean ± SEM; n=12 mice/group.


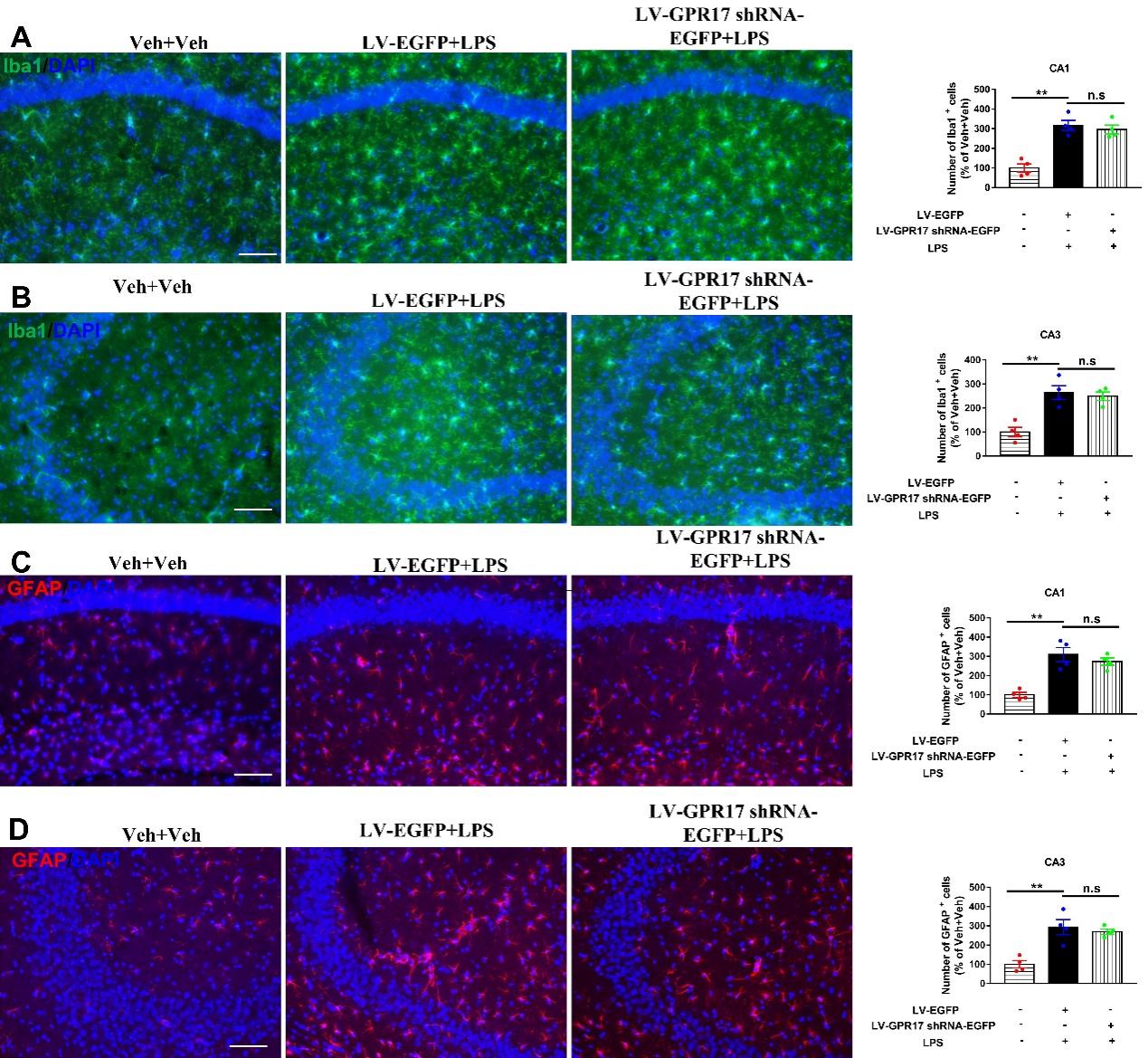


**Fig. S4. GPR17 knockdown had no significant effect on the expression of glial cells in the hippocampal CA1 and CA3 regions.** Representative images of immunofluorescent staining of Iba1 (green) and DAPI (blue) in the hippocampal (A) CA1 and (B) CA3 region of lentivirus pretreatment mice. The number of Iba1 antibody-stained microglia, in the hippocampal CA1 and CA3 region of LV-GPR17-shRNA pretreatment mice. Representative images of immunofluorescent staining of GFAP (red) and DAPI (blue) in the hippocampal (C) CA1 and (D) CA3 region of lentivirus pretreatment mice. The number of GFAP antibody-stained astrocytes, in the hippocampal CA1 and CA3 region of lentivirus pretreatment mice.Values shown are expressed as mean ± SEM; n=4 mice/group. *** P <0.01* versus Control group. Scale bars = 100 μm.


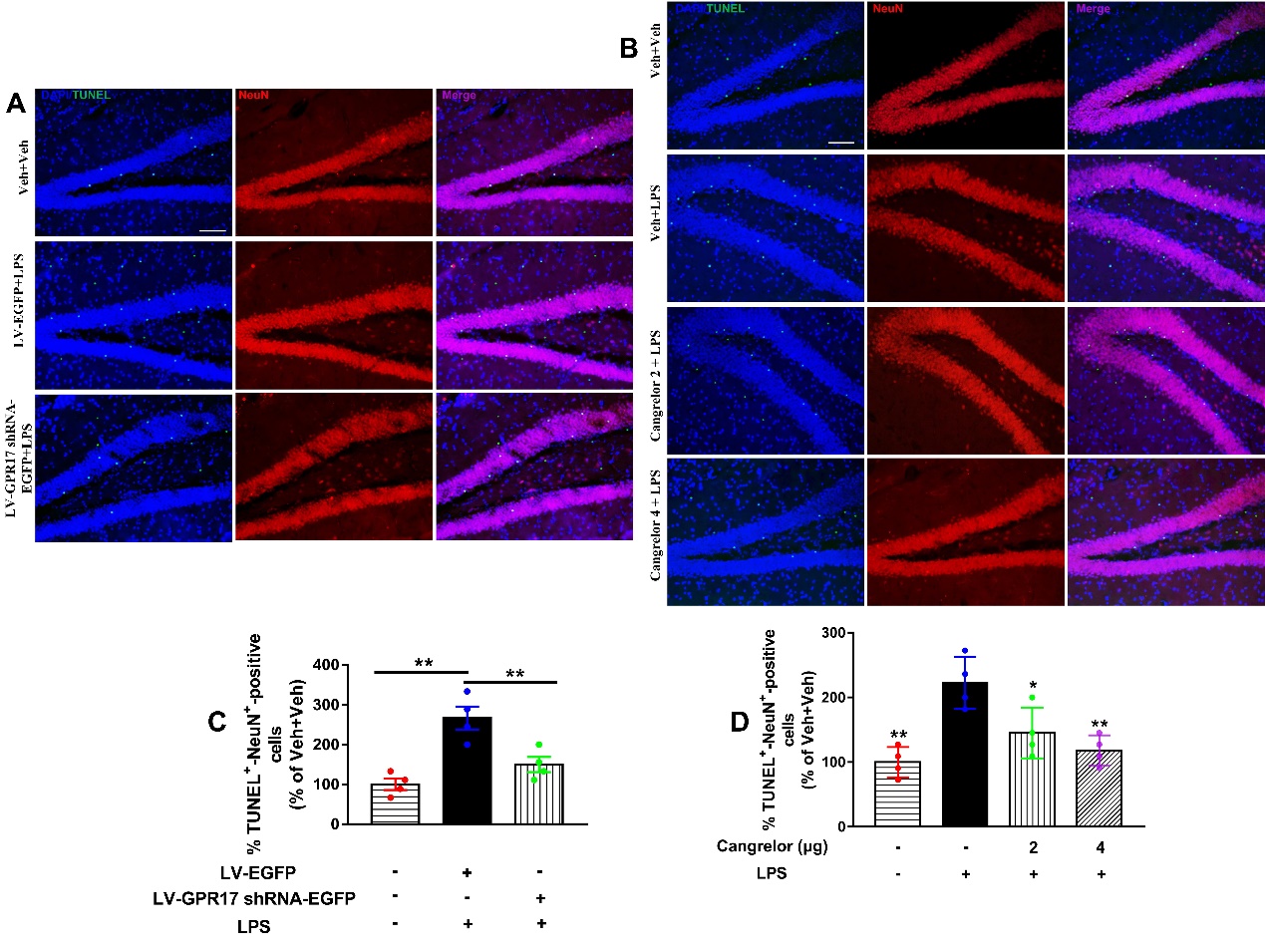


**Fig. S5.** **Knockdown and inhibition of hippocampal GPR17 ameliorated neuron apoptosis in LPS-treated mice.** (A, B) TUNEL assay and DAPI nuclear stain were used to identify dead cell nuclei. TUNEL assay (green) was performed with immunofuorescence for NeuN (red), and DAPI (blue). TUNEL-positive nuclei were co-localized in NeuN-positive cells in the hippocampus. (C, D) Quantitative analysis of TUNEL^+^-NeuN^+^-positive cells. Values shown are expressed as mean ± SEM; n=4 mice/group. **P<0.05, ** P <0.01* versus Veh+LPS. Scale bar, 100 μm.


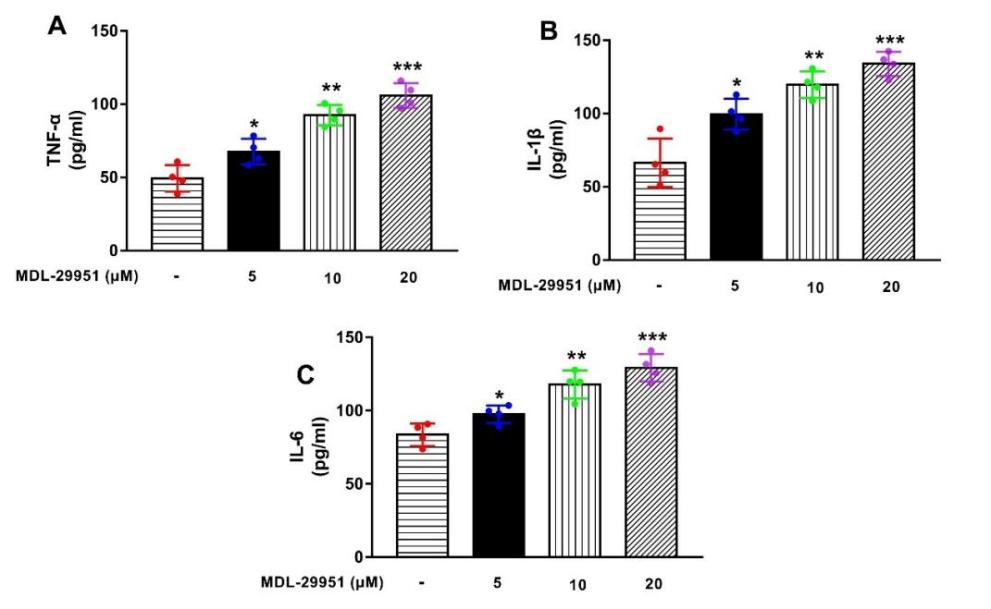


**Fig. S6.** **MDL-29951 treatment increased inflammatory cytokines in BV-2 cells.**

The cells were pretreated with MDL-29951 (5,10, or 20 µM), Then, culture supernatant was collected to detect the production of TNF-α (A), (B) IL-1β, and IL-6 (C). Data shown are expressed as mean ± SEM; n = 4. **P<0.05, ** P <0.01, *** P <0.001* versus Control group.
